# Endothelial Dysfunction in Heart Failure With Preserved Ejection Fraction: What are the Experimental Proofs?

**DOI:** 10.3389/fphys.2022.906272

**Published:** 2022-07-08

**Authors:** Lauriane Cornuault, Paul Rouault, Cécile Duplàa, Thierry Couffinhal, Marie-Ange Renault

**Affiliations:** Univ. Bordeaux, INSERM, Biology of Cardiovascular Diseases, U1034, Pessac, France

**Keywords:** heart failure, diastolic dysfunction, endothelial cells, cardiomyocytes, animal models, pathophysiology, intercellular crosstalk

## Abstract

Heart failure with preserved ejection fraction (HFpEF) has been recognized as the greatest single unmet need in cardiovascular medicine. Indeed, the morbi-mortality of HFpEF is high and as the population ages and the comorbidities increase, so considerably does the prevalence of HFpEF. However, HFpEF pathophysiology is still poorly understood and therapeutic targets are missing. An unifying, but untested, theory of the pathophysiology of HFpEF, proposed in 2013, suggests that cardiovascular risk factors lead to a systemic inflammation, which triggers endothelial cells (EC) and coronary microvascular dysfunction. This cardiac small vessel disease is proposed to be responsible for cardiac wall stiffening and diastolic dysfunction. This paradigm is based on the fact that microvascular dysfunction is highly prevalent in HFpEF patients. More specifically, HFpEF patients have been shown to have decreased cardiac microvascular density, systemic endothelial dysfunction and a lower mean coronary flow reserve. Importantly, impaired coronary microvascular function has been associated with the severity of HF. This review discusses evidence supporting the causal role of endothelial dysfunction in the pathophysiology of HFpEF in human and experimental models.

## Introduction

A significant proportion of patients with clinical syndrome of heart failure (HF) happen to have a normal left ventricular ejection fraction (EF) referred to as heart failure with preserved ejection fraction (HFpEF) as opposed to patients with reduced ejection fraction (HFrEF). The clear lesson from the past years is that making a firm diagnosis of chronic HFpEF remains a challenge. The European Society of Cardiology recommend a stepwise diagnostic process, first step is clinical, symptoms signs and demographics, laboratory tests, electrocardiogram, the second step is echocardiography and Natriuretic Peptide Score, step 3 is echocardiographic or invasive haemodynamic exercise stress tests and step 4 is the final etiology (Pieske et al., 2020; Nagueh 2021). HFpEF is characterized by increased arterial and myocardial stiffness and decreased left ventricular (LV) relaxation which causes increased LV end-diastolic pressure with impaired LV filling. This diastolic dysfunction is associated with abnormal ventricular-arterial coupling, pulmonary hypertension, chronotropic incompetence and cardiac reserve dysfunction (Zhou et al., 2021). Notably, HFpEF now accounts for more than 50% of all HF patients and has turned into the most common HF phenotype (Tsao et al., 2018). However, in contrast to HFrEF, for which significant advances in understanding etiologies and mechanisms have been made, HFpEF pathophysiology is still poorly understood (Lyle and Brozovich, 2018). HFpEF patients are generally older, more often females, with a high prevalence of cardiovascular and non-cardiovascular comorbidities, such as obesity, metabolic syndrome, type 2 diabetes mellitus, hypertension, atrial fibrillation or renal dysfunction (Groenewegen et al., 2020). At molecular and cellular level HFpEF has been associated with cardiac fibrosis, cardiomyocyte hypertrophy and stiffness, microvascular dysfunction, inflammation, decreased bioavailability of NO and oxidative stress (Zhou et al., 2021). Accordingly, in 2013, WJ Paulus and C Tschöpe proposed a paradigm for HFpEF pathophysiology: cardiovascular risk factors lead to a systemic low-grade inflammation that would trigger endothelial cells (EC) and coronary microvascular dysfunction; in turn, this small vessel disease would be responsible for cardiac wall stiffening and diastolic dysfunction (Paulus and Tschöpe, 2013). This paradigm is essentially based on the fact that vascular and microvascular dysfunction is highly prevalent in HFpEF patients (Tona et al., 2021). It is then reasonable to make three hypotheses: (1) Cardiac EC dysfunction (modification of EC properties) causes diastolic dysfunction, (2) diastolic dysfunction is initiated by cardiomyocyte impairment which subsequently induces ED and (3) the 2 phenomenon’s, i.e., endothelial dysfunction and diastolic dysfunction, occur concomitantly but are not interconnected.

This review will discuss current knowledge supporting that modifications of EC properties may affect cardiomyocyte homeostasis and cardiac function. Importantly, in this revue endothelial dysfunction will not only design endothelium-dependent vasodilation but any detrimental modification of the endothelial phenotype. Notably, it will also design impaired endothelial barrier properties, endothelial activation, impaired endothelial paracrine activity, impaired endothelial survival, impaired endothelial quiescence. Endothelial dysfunction may be measured via multiple techniques including noninvasive technics in patients (Theodorakopoulou et al., 2021; Xu et al., 2022), histological analysis in animal models and cell culture assays (Bednarek 2022).

## Subsections Relevant for the Subject

### Evidence of Cardiac Microvascular Dysfunction in HFpEF Patients

At first, patients with HFpEF were shown to have impaired systemic endothelial function, this was evidenced by reactive hyperemia peripheral arterial tonometry (Akiyama et al., 2012) and confirmed in 2016 by measuring brachial artery flow mediated dilation (Marechaux et al., 2016). Importantly in this study, the authors revealed that HFpEF is not only associated with “large vessel” dysfunction but also with microvascular dysfunction measured in the skin of the forearm using a laser Doppler flow probe (Marechaux et al., 2016). More recently, a videodermatoscope was used to image nailfold capillaries and revealed that the number of patients with abnormal video capillaroscopic findings was significantly greater in the HFpEF group compared to HFrEF and control groups (Yüksel et al., 2021). In addition to systemic vascular dysfunction, 70–75% of HFpEF patients were shown to have lower coronary myocardial flow reserve. This was measured via both non-invasive and invasive imaging techniques including echography and angiography (TIMI frame count and myocardial blush grade) (Sucato et al., 2015), cardiac positron emission tomography (Srivaratharajah et al., 2016) cardiac magnetic resonance (Kato et al., 2016; Löffler et al., 2019), transthoracic Doppler echocardiography (Shah et al., 2018) and hemodynamic measures during cardiac catheterization (Dryer et al., 2018). Cardiac catheterization also revealed that HFpEF patients have a higher mean index of microvascular resistance compared to controls (Dryer et al., 2018).

The PROMIS-HFpEF trial demonstrated a high prevalence of microvascular dysfunction in HFpEF, as evaluated by the coronary flow reserve, in the absence of unrevascularized macrovascular coronary artery disease. The microvascular dysfunction was correlated with systemic endothelial dysfunction (as well as markers of HF severity (Shah et al., 2018). More specifically, among the HFpEF patients presenting a microvascular coronary disease (72%), 29% were shown to have endothelium dependent microvascular dysfunction (acetylcholine-induced vasodilation) and 33% to have endothelium independent microvascular dysfunction (adenosine-induced vasodilation) (Yang et al., 2020). Notably, patients showing endothelium-independent microvascular dysfunction had worse diastolic dysfunction (E/e’) and higher overall mortality**.** Anyways, cardiac magnetic resonance imaging has revealed that 96% of patients with HFpEF demonstrate at least a regional oxygenation impairment (Fischer et al., 2022).

Cardiac magnetic resonance imaging showed that 83% of patients with HFpEF patients display increased extracellular volume (T1 mapping) reflecting either high water content and/or increased fibrotic load (Löffler et al., 2019; Fischer et al., 2022). T2 mapping suggested 66% of patients actually have myocardial edema. In Fischer et al. study, attenuated myocardial oxygenation reserve was associated with myocardial edema and diastolic dysfunction (Fischer et al., 2022).

Evidence of microvascular disease in patients with HFpEF has been verified by histological analysis performed on heart biopsies. First, cardiac biopsies from patients with HFpEF obtained post mortem, have revealed that HFpEF patients have decreased cardiac microvascular density and diffused fibrosis (Mohammed et al., 2015). In myocardium biopsies of HFpEF patients, Franssen et al. (2016) reported that E-Selectin (SELE) and Intercellular Adhesion Molecule-1 (ICAM-1) expression levels were upregulated; NADPH Oxidase 2 (NOX2) expression was raised in macrophages and ECs but not in cardiomyocytes; and that Nitric Oxide Synthase 3 (NOS3) uncoupling, which was associated with reduced myocardial nitrite/nitrate concentration, cyclic guanosine monophosphate (cGMP) content, and Protein Kinase cGMP-Dependent 1 (PRKG1) activity. Another study performed on skin biopsies from HFpEF patients confirmed the rarefaction of blood capillaries but also revealed that HFpEF patients display a reduced lymphatic vessel density associated with diminished expression of lymphatic markers lymphatic vessel endothelial hyaluronan receptor 1 (LYVE1), Prospero Homeobox Protein 1 (PROX1) and Vascular Endothelial Growth Factor C (VEGFC) (Rossitto et al., 2020).

Accordingly, WJ Paulus’s group proposed that in HFpEF, decreased NOS3 activity in ECs would decrease nitric oxide (NO) available to activate Soluble Guanylate Cyclase (sGC) in cardiomyocyte leading to decreased cGMP production, PRKG1 activity and subsequently Titin phosphorylation, ultimately leading to cardiomyocyte stiffening. In this paradigm, impaired NOS3 activity is proposed to be induced by low-grade inflammation associated with cardiovascular risk factor (Paulus and Tschöpe, 2013).

### How Can EC Dysfunction Cause Cardiomyocyte Impairment And/Or Cardiac Dysfunction?

**FIGURE 1:**
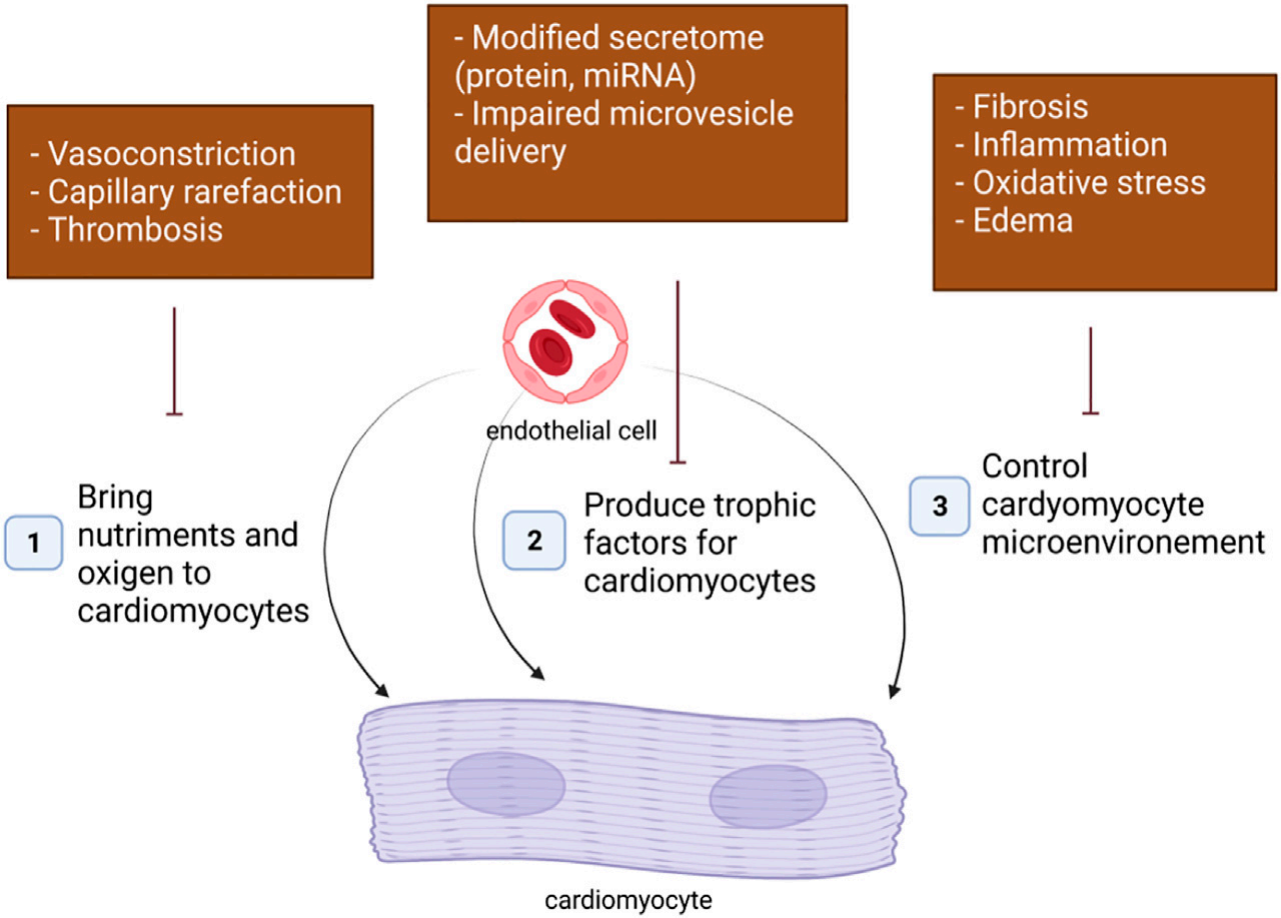
Endothelial cells may affect cardiomyocyte homeostasis in many ways.

In adults, ECs may affect cardiomyocyte function in many ways (Figure 1).

1. The first essential role of EC as a cell component of blood vessels is to bring nutriments and oxygen to cardiomyocytes. Cardiac hypoxia and subsequent cardiomyocyte dysfunction may then be a consequence of an impaired regulation of vascular tone occurring at the arteriole levels, a capillary rarefaction due to impaired angiogenesis or increased endothelial apoptosis and a vascular obstruction following thrombosis.
2. Besides, ECs were shown to produce signals necessary for cardiomyocyte homeostasis including NO, Endothelin 1 (EDN1), Apelin (APLN). An alteration of EC secretome may then also modify cardiomyocyte properties.
3. Finally, ECs dysfunction may affect cardiomyocyte function indirectly by modifying cardiomyocyte microenvironment. Notably, EC dysfunction may promote cardiac fibrosis, cardiac inflammation or cardiac edema via increased production of pro-fibrotic, pro-inflammatory signals or impaired barrier properties.

#### Impaired Nutriment/O_2_ Delivery

##### Impaired Vasomotricity

Impaired endothelium-dependent vasodilation is the most studied feature of endothelial dysfunction. As described above, it has been widely associated with diastolic dysfunction and suggested to play a critical role in cardiac perfusion. NO produced by the endothelial isoform of NO synthase (NOS3) in ECs is considered to be the central regulator of vascular tone (Leo et al., 2021). Mechanistically, NO diffuses from ECs to smooth muscle cells (SMCs) to activate sGC promoting SMC relaxation and subsequent vasodilation of arterioles (Farah et al., 2018). Accordingly, chronic administration of the nitroxyl donor 1-nitrosocyclo hexyl acetate (1-NCA) was shown to increase coronary flow *ex vivo* and to limit left ventricular diastolic dysfunction attested by increased E/A and decreased isovolumic relaxation time (IVRT) in streptozotocin-induced diabetic mice (Cao et al., 2015). This was associated with a decreased cardiomyocyte size, a decreased Myosin Heavy Chain 7 (MYH7) expression and a decreased pro-fibrotic signal level (Connective Tissue Growth Factor (CTGF)). However, considering the wide range of action of NO (Farah et al., 2018) the specific contribution of impaired endothelium dependent vasodilation is hard to quantify.

Besides, myocardial capillaries, but not arterioles have been demonstrated to be the primary determinant of coronary flow reserve. Indeed, contribution of capillaries to total myocardial vascular resistance was shown to be 75% during hyperemia (Jayaweera et al., 1999) suggesting that arterioles of which the dilation depends on the endothelium only account for 25% myocardial flow reserve during hyperemia. The effect of NO on cardiac pericyte which cover cardiac capillaries is actually not known.

##### Capillary Rarefaction/Impaired Angiogenesis

The relationship between capillary density and cardiomyocyte phenotype has been widely investigated in the setting of physiological/adaptive cardiac hypertrophy. Angiogenesis was shown to be necessary for cardiomyocyte growth/hypertrophy. Notably, overexpression of proangiogenic factors including the peptide regulator of angiogenesis (PR39) (Tirziu et al., 2007) and Placental Growth Factor (PGF) (Jaba et al., 2013) in the heart were shown to induce cardiac hypertrophy. Consistently, endothelial specific disruption of Fms Related Receptor Tyrosine Kinase 1 (*Flt1*), simulating Kinase Insert Domain Receptor (KDR) downstream signaling in the myocardium was shown to increase cardiac capillary density and to promote cardiomyocyte hypertrophy in adult mice (Kivelä et al., 2019). In this study, ECs were proposed to induced cardiomyocyte hypertrophy via Neuregulin 1 (NRG1) overexpression (Kivelä et al., 2019). Conversely, cardiomyocyte Vascular Endothelial Growth Factor A (*Vegfa*) gene deletion, decreased capillary density and induced slight cardiac atrophy (Giordano et al., 2001). More strikingly, when cardiac hypertrophy was provoked either by transverse aortic constriction (TAC) (Izumiya et al., 2006) or AKT Serine/Threonine Kinase 1 (AKT1) overexpression (Shiojima et al., 2005) in cardiomyocytes, inhibiting angiogenesis by soluble FKL1 was shown to prevent cardiac hypertrophy. Altogether demonstrating that angiogenesis is necessary for adaptive cardiomyocyte hypertrophy.

However, the imbalance between capillary density and myocardial fiber growth is suggested to be an important contributor to the transition from hypertrophy to HF (Shiojima et al., 2005). Since blocking VEGF-induced signaling by soluble FLK1 in mice not only prevented cardiomyocyte hypertrophy but also decreased fractional shortening (FS), exacerbated LV end-diastolic pressure (EDP) elevation and increased cardiac fibrosis (Shiojima et al., 2005; Izumiya et al., 2006).

##### Thrombosis

The contribution of thrombosis associated with large vessel atherosclerosis has been widely investigated in ischemic heart disease (Al Said et al., 2018). However, little is known about small vessel thrombosis which may lead to capillary occlusion. Recent experimental studies suggest that small vessel thrombosis may contribute to HF. Increased thrombosis following neutrophils activation was shown to exacerbate Angiotensin-2 (ANG2)-induced cardiac hypoxia, cardiomyocyte hypertrophy and diastolic dysfunction (Tang et al., 2021). Treatment with recombinant ADAM Metallopeptidase With Thrombospondin Type 1 Motif 13 (ADAMTS13), which cleaves Von Willebrand Factor (VWF) and inhibits its adhesion properties, prevents ascending aortic constriction (AAC)-induced cardiac capillary thrombosis, cardiac fibrosis and improved systolic function (increased EF) (Witsch et al., 2018). Finally, P-selectin was shown to increase angiotensin II-induced cardiac inflammation and fibrosis via platelet activation using P-selectin deficient mice (Liu et al., 2016).

#### Impaired Paracrine Function, EC-Cardiomyocyte Crosstalk

Intercellular cross-talks are proposed to play substantial modulatory roles in the normal and failing heart of adults. More specifically, factors secreted by cardiac microvascular ECs, or transported via extracellular vesicle from ECs to cardiomyocytes, were shown to modulate cardiac performance and to affect cardiac remodeling either positively or negatively (Segers et al., 2018). Accordingly, co-culturing cardiomyocyte with EC was shown to promote cardiomyocyte contraction and relaxation (Homme et al., 2021). Besides endothelial derived exosomes were shown to protect cardiomyocytes from apoptosis upon hypoxic or inflammatory or high glucose conditions (Hu et al., 2018; Cao et al., 2021; Liu et al., 2022).

The role of NO, APLN, EDN1, NRG1, reactive oxygen species (ROS) and miRNA which have been extensively studied is discussed below.

##### Nitric Oxide

**FIGURE 2:**
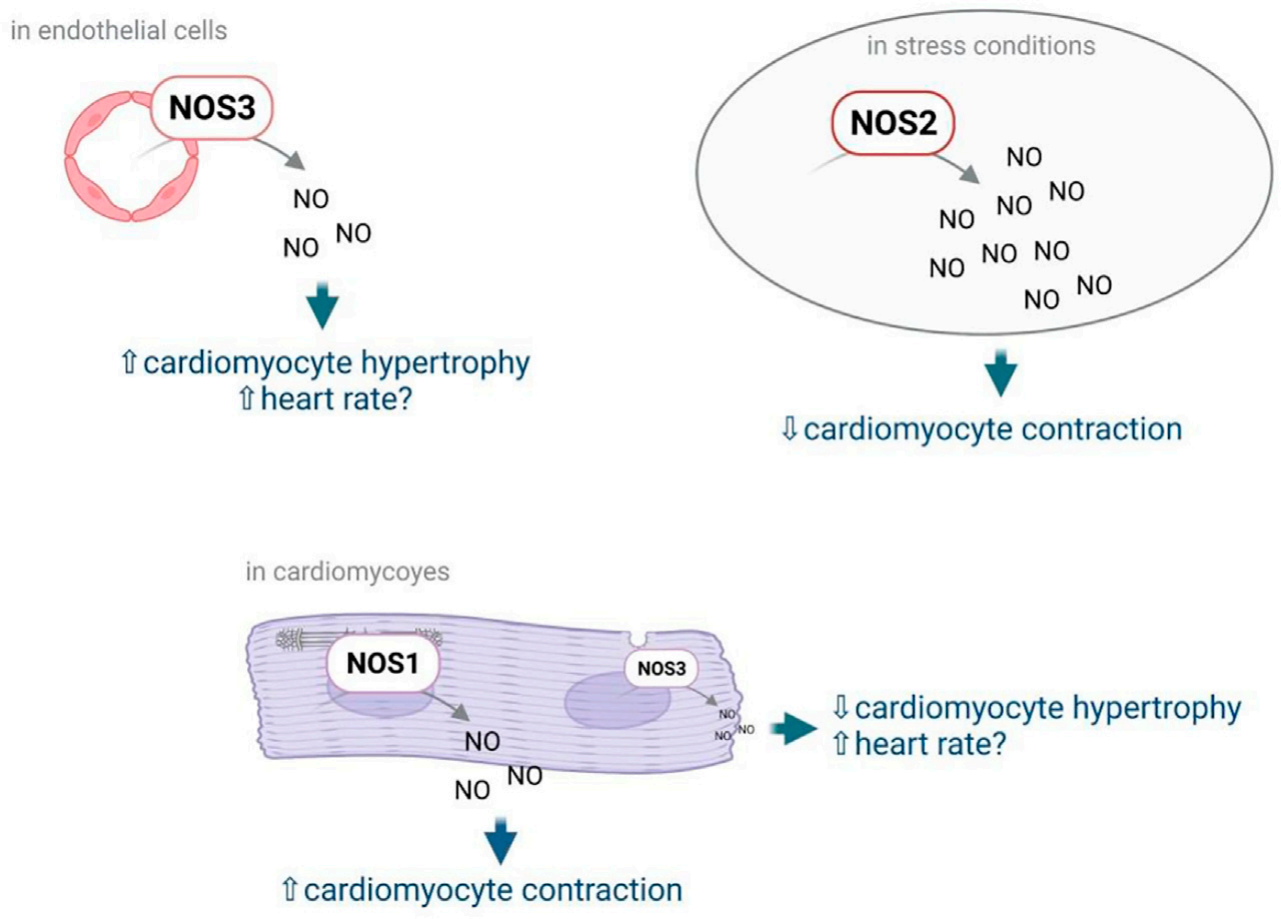
NO effects on cardiomyocytes depend on the NOS by which it has been produced, and the cells in which it has been produced. Notably NO produced by NOS3 in ECs may promote cardiomyocyte hypertrophy while NO produced by NOS3 in cardiomyocytes was shown to prevent cardiomyocyte hypertrophy. NO produced by NOS3 was also shown to increase heart rate; however the cell type in which NO is produced is not known. NO produced by NOS1 in cardiomyocytes promotes cardiomyocyte contractility while NO produced by NOS2 upon stress decreases cardiomyocyte contractility.

NO is the most studied EC mediator. Mainly known for its vasodilator effect, NO has also direct effects on cardiomyocytes. NO modulate cardiomyocyte contractility and heart rate but also cardiomyocyte growth and stiffness (Rastaldo et al., 2007). In the heart, NO is not only produced by NOS3 but also by the neuronal NOS (or NOS1) and the inducible NOS (NOS2). NOS3 is expressed predominantly in coronary vascular and endocardial ECs. At a much lower level, it is also detected in cardiac myocytes and sinoatrial and atrioventricular nodal cells. NOS1 is expressed in pre- and postganglionic fibers innervating the sinoatrial and atrioventricular nodes, in subepicardial neuronal cells, and in intrinsic cardiac neurons. Besides, NOS1 is also found in the sarcoplasmic reticulum of cardiac myocytes. NOS2 can be expressed in infiltrating inflammatory cells, coronary microvascular and endocardial ECs, coronary vascular smooth muscle, fibroblasts, and cardiac myocytes, depending upon the stimulus (Shah and MacCarthy, 2000) (Figure 2). Importantly, NOS3 and NOS1 do produce low levels of NO, while NOS2 produces high levels of NO (Rastaldo et al., 2007). NO signals by activating the sGC which produce cGMP, high cGMP levels do activate PRKG1, while low cGMP levels do inhibit Phosphodiesterase 3 leading to increased cAMP levels and Protein Kinase cAMP-Activated (PRKA) activation. Alternatively, NO may promote S-nitrosylation of various proteins including Ryanodine Receptor 2 (Rastaldo et al., 2007) but also myocardial G protein-coupled receptor (GPCR) signaling components, GPCR kinases (GRKs) and β-arrestins (Kayki-Mutlu and Koch, 2021).

The effect of NO on **cardiomyocyte contractility** depends on NO levels, low levels of NO have positive inotropic effects while high levels of NO do have negative inotropic effects. Notably, regulation of cardiomyocyte contractility actually depends on NOS1- but not NOS3-derived NO (Martin et al., 2006). In cardiomyocytes, signaling via NOS1 and NOS3 is compartmentalized and each isoform modulates cardiac function differently. NOS3 is localized to the caveolae and blunts the response to β-adrenergic stimulation via inhibition of the L-type Ca2+ current, while NOS1 is localized to the sarcoplasmic reticulum and enhances contraction by allowing more Ca2+ to exit the sarcoplasmic reticulum (Ziolo et al., 2008) (Ally et al., 2020).

On the contrary, regulation of **cardiomyocyte growth** seems to mainly depend on NOS3. Indeed *Nos3* KO mice just like triple *Nos1*, *Nos2*, *Nos3* KO mice develop cardiac hypertrophy. NOS1 and NOS2 may also slightly prevent cardiac hypertrophy since hypertrophy is significantly higher in triple KO mice in comparison to *Nos3* KO mice, however, *Nos*1 or *Nos*2 KO mice do not develop cardiac hypertrophy (Tsutsui et al., 2015). Accordingly, stimulation of NOS3 transcription with AVE3085 was shown to prevent cardiomyocyte hypertrophy in dahl salt-sensitive rats. Consistently, the NO donor, LA419, was shown to prevent cardiomyocyte hypertrophy induced by the aortic stenosis technique in rats (Ruiz-Hurtado et al., 2007). However, *in vitro* studies performed using freshly isolated adult ventricular cardiomyocytes suggest that regulation of cardiomyocyte hypertrophy more likely depends on NOS3 from cardiomyocytes (Wenzel et al., 2007). Consistently, cardiomyocyte-specific overexpression of NOS3 was shown to reduce compensatory hypertrophy after myocardial infarction (Janssens et al., 2004). Endothelial NOS3 may actually promote cardiomyocyte hypertrophy since L-NAME was shown to prevent cardiomyocyte hypertrophy upon administration of pro-angiogenic factors (Tirziu et al., 2007; Jaba et al., 2013). Besides, stimulation of sGC with BAY 41-8543 was shown to improve HFpEF in rats harboring the human Renin and human Angiotensinogen genes (dTGR); however, it did not prevent hypertrophy (Wilck et al., 2018) suggesting that NOS3 regulation of cardiomyocyte growth does not depend on sGC activation by NO.

**Regulation of heart rate** also depends on NOS3 since *Nos3* KO mice but not *Nos1* or *Nos2* KO mice display reduced heart rate (Tsutsui et al., 2015). It is not known whether regulation of heart rate relies of endothelial or cardiomyocyte NOS3.

NO was suggested to prevent **cardiomyocyte stiffness** by regulating Titin phosphorylation via PRKG1 and/or PRKA activation. Humans HF is associated with increased passive stiffness, decreased cGMP and PRKG1 activity (Borbély et al., 2009; van Heerebeek et al., 2012). Moreover, administration of PRKA and PRKG1 was shown to phosphorylate Titin (Krüger et al., 2009) and to decrease cardiomyocyte stiffness *in vitro* (Borbély et al., 2005; Borbély et al., 2009). The identity of the NOS regulating cardiomyocyte stiffness is not known. In mice, only triple *Nos1*, *Nos2*, *Nos3* KO mice display diastolic dysfunction (E/A wave ratio ≥1.5, decreased dP/dt min and increased Tau), with preserved left ventricular systolic function (Tsutsui et al., 2015). Nevertheless, administration of AVE3085 promoting NOS3 transcription did not modify Titin phosphorylation in Dahl salt-sensitive rats (Westermann et al., 2009).

In summary, there is actually no evidence showing that NO produced in ECs by NOS3 may modify cardiomyocyte phenotype. Endothelial specific KO of *Nos3* would be necessary to demonstrate such phenomenon. Besides, not all study agrees on the cardiac phenotype of *Nos3* deficient mice, while two studies showed that these mice develop diastolic dysfunction attested by increased EDP (Vignon-Zellweger et al., 2011; Vignon-Zellweger et al., 2014), two other studies show that EDP was not modified in these mice (Tsutsui et al., 2015; Ebner et al., 2017).

##### Endothelin

EDN1 has been first isolated from aortic ECs and was shown to have inotropic and chronotropic effects on cardiomyocytes (Hu et al., 1988; Concas et al., 1989). Also, it may also promote cardiomyocyte hypertrophy (Ito et al., 1991). EDN1 was confirmed to be expressed by vascular and endocardial ECs (Giaid et al., 1995), but also shown to be expressed by smooth muscle cells (Peter and Davenport, 1995) and cardiomyocytes (Giaid et al., 1995). Cardiomyocyte or specific endothelial deletion of *Edn1* (Heiden et al., 2014) demonstrated that cardiomyocyte hypertrophy was regulated by cardiomyocyte-derived EDN1 (Shohet et al., 2004) but not endothelial-derived EDN1 (Heiden et al., 2014).

##### Neuregulin 1

NRG1 which is expressed by vascular and endocardial ECs has been proposed to signal to cardiomyocytes which express NGR1 receptors, Erb-B2 Receptor Tyrosine Kinase 2 (ERBB2) and Erb-B2 Receptor Tyrosine Kinase 4 (ERBB4). Soluble recombinant NRG1 was shown to promote cardiomyocyte survival (Liu et al., 2006) and hypertrophy (Zhao et al., 1998; Baliga et al., 1999) after LAD ligation or pacing-induced HF. Accordingly, endothelial specific deletion of *Nrg1* increased infarct size after left descending coronary artery ligation (Hedhli et al., 2011). However, unexpectedly, cardiomyocyte specific deletion of *Erbb2* was shown to induce cardiomyocyte hypertrophy (Ozcelik et al., 2002), while endothelial specific disruption of *Erbb4* was shown to prevent transverse aortic constriction induced cardiac hypertrophy and fibrosis (Dugaucquier et al., 2020). These results suggest that NRG1 does not directly signal to cardiomyocyte but exert its “cardiac” action via autocrine signaling in ECs. Besides, endothelial derived NRG1 was shown to promotes ischemia-induced angiogenesis and arteriogenesis in the setting of hind limb ischemia (Hedhli et al., 2012) which may explains the deleterious effect of endothelial specific KO of *Nrg1* in the setting of myocardial infarction.

##### Apelin

APLN is an important paracrine regulator of cardiovascular pathophysiology and acts through binding to its GPCR Apelin Receptor (APLNR). In the heart, APLN was shown to be secreted by endocardial and vascular ECs (Kleinz and Davenport., 2004) and to bind to APLNR in cardiomyocytes, vascular SMCs, and ECs (Kleinz et al., 2005). Activation of APLNR by APLN was shown to promote cardiomyocyte contractility (Szokodi et al., 2002; Farkasfalvi et al., 2007). Besides, APLN was shown to have cardio-protective effects upon ischemia reperfusion injury (Simpkin et al., 2007). Finally, infusion of APLN was shown to protect from cardiac hypertrophy (Scimia et al., 2012) by lowering systolic pressure and or preventing stretch-induced cardiomyocyte hypertrophy. However, unexpectedly, both *Aplnr* constitutive KO (Scimia et al., 2012) and *Aplnr* cardiomyocyte specific KO (Parikh et al., 2018) were shown to be protected from TAC-induced cardiomyocyte hypertrophy. This effect is opposite and independent on APLN. Indeed, cardiomyocyte APLNR is proposed to be activated by mechanical stretch and to participate in mechanical stretch-induced cardiomyocyte hypertrophy (Scimia et al., 2012). Notably, TAC-induced hypertrophy is exacerbated in endothelial-specific *Aplnr* KO consistent with the anti-hypertrophic role of APLN (Parikh et al., 2018).

##### Reactive Oxygen Species

The main producers of ROS in blood vessels are activated pro-oxidative enzymes, such as NADPH oxidase (NOX), Xanthine oxidase and uncoupled NOS. Notably, NOS coupling is dependent on sufficient availability of substrate (L-arginine), tetrahydrobiopterin, and cofactors as well as an equilibrated redox environment. Deficiency in any of these components will induce NOS uncoupling, with electrons shuffling to dioxygen (O_2_) and production of superoxide (O_2_^−^) instead of NO (Farah et al., 2018). However, increasing evidence points out to the growing role of mitochondrial and endoplasmic reticulum enzymes. Their activity is regulated by many factors, including humoral factors (e.g., cytokines) and physical factors (e.g., stretching). Among ROS, we can distinguish such reactive forms as O_2_^−^, hydrogen peroxide (H_2_O_2_), singlet oxygen, hydroxyl radicals, peroxyl radicals, alkoxyl radicals, peroxynitrite, hypochlorous acid and ozone. In the heart, ROS may activate Calcium/Calmodulin Dependent Protein Kinase II (CAMK2) leading to excitation-contraction coupling but also Mitogen-Activated Protein Kinase 14 (MAPK14) and Mitogen-Activated Protein Kinase 8 (MAPK8) activation leading to inhibition of Insulin signal transduction. ROS may also activate PRKA, or transcription factors such as Nuclear Factor Kappa B (NF-κB). Notably activation of CAMK2 and MAPK14 in cardiomyocyte is pro-apoptotic. Besides ROS are proposed to promote cardiomyocyte hypertrophy (Dubois-Deruy et al., 2020). While ROS are suggested to be mainly produced by cardiomyocytes in the setting of HFrEF, they may be produced by ECs in the setting of HFpEF (Münzel et al., 2015). There is actually very little evidence that endothelial-derived ROS may promote cardiomyocyte impairment and cardiac dysfunction. Nevertheless, one study has shown that endothelial NOX2 overexpression exacerbated ANG2-induced diastolic dysfunction (decreased end-diastolic volume, increased LV diastolic stiffness and EDP). This was associated with increased cardiac fibrosis and inflammation. Capillary density was not modified (Murdoch et al., 2014). Notably, endothelial NOX2 overexpression alone was not sufficient to induce cardiac fibrosis or inflammation. On the contrary, another study shows that NOX4 overexpression in ECs was shown to increase cardiac output both upon physiological conditions and ANG2 infusion (Wang et al., 2021). This was associated with cardiac hypertrophy, but decreased cardiac fibrosis and inflammation. Besides, mice overexpression NOX4 were shown to have enhanced endothelium-dependent vasodilation which is mediated by increased H_2_O_2_ levels (Ray et al., 2011, 4).

Altogether these results demonstrate that the effect of endothelial-derived ROS is far from being fully understood.

##### miRNAs

miRNA were also shown to participate in the dialogue from ECs to cardiomyocytes by transiting in exosomes or extracellular vesicles. Notably, miR-126 and miR-210, produced in ECs overexpressing HIF1A were shown to be transferred to cardiomyocyte via micro-vesicles in which they activate pro-survival kinases and induce a glycolytic switch (Ong et al., 2014).

HUVECs were shown to produce exosomes able to prevent cultured cardiomyocyte cells death (Davidson et al., 2018). Also MiR-126 included in extracellular vesicles from ECs was shown to prevent cardiac hypertrophy (Chen et al., 2017). Finally, Mir-146a of which expression was shown to be increased in ECs stimulated by prolactin, was shown to be transferred into cardiomyocytes and to reduce expression of Erbb4, Notch1, and Irak1 and to decrease metabolic activity (Halkein et al., 2013)**.**

##### Other EC-Derived Mediators

A long list of molecules produced by ECs and known to modulate the phenotype of cardiomyocytes are proposed to participate in the dialogue between ECs and cardiomyocytes including Interleukin-6, Periostin, Tenascin-C, Thrombospodins, Follistatin-like 1, Frizzled-related protein 3, Insulin-like growth factor-1, Connective tissue growth factor, Dickkopf-3, Bone morphogenetic protein-2 and -4, Interleukin-1β, Placental growth factor, Leukemia inhibitory factor, Wnt1-induced secreted protein-1, Midkine, Adrenomedullin (Segers et al., 2018) and VEGFB (Balberova et al., 2021). However, whether the molecules listed above are actually acting on cardiomyocyte to induce the observed effects remains to be demonstrated using cell specific conditional KO mice.

##### Conclusion

In summary, the proposed role of NO, NRG1, APLN and EDN1 in mediating endothelial to cardiomyocyte signaling is more likely not true since these molecules mainly acts autocrinally. Indeed the NO modulating the phenotype of cardiomyocyte is mainly produced by cardiomyocytes themselves. Cardiomyocyte hypertrophy is regulated by cardiomyocyte- but not endothelial-derived EDN1 (Shohet et al., 2004). NRG1 does not directly signal to cardiomyocyte but exert its “cardiac” action via autocrine signaling in ECs (Dugaucquier et al., 2020). Finally, APLN exert its anti-hypertrophic effect by signaling to ECs themselves. Notably the importance of autocrine signaling in the heart has been recently reviewed (Segers and De Keulenaer, 2021).

The molecular mediator of endothelial to cardiomyocyte signal remains to be identified.

#### Modifying the Cardiomyocyte Microenvironment

##### Capillary Leakage/Edema

Capillary leakage leads to cardiac edema and alters cardiac physiology. Myocardial fluid homeostasis is determined by the balance between the fluid filtration rate from the coronary microvasculature into the interstitium and the lymphatic removal rate of fluid from the interstitium. Importantly, cardiac edema may disturb cell-cell contacts, cardiac wall elasticity and/or compress cardiac capillaries. Notably, it has been shown that an only 2% accumulation of fluid in the cardiac interstitium can induce a rise in the interstitial pressure and result in cardiac wall stiffening impairing cardiac relaxation. Besides, as fluid accumulates within the interstitium, the diffusion distance for O_2_ between the capillaries and myocytes increases, this is critical for cardiomyocytes which operate with a near-maximum oxygen extraction capacity at all times. Unfortunately, experimental proofs of such phenomena are difficult to obtain.

However, several studies have highlighted the association between abnormal vascular leakage and/or impaired lymphatic drainage and cardiac dysfunction. Notably, Src blockade which stabilizes a FLK1/Cadherin 5 (CDH5) complex was shown to reduce edema and tissue injury following myocardial infarction (Weis et al., 2004), VEGF-C/VEGFR-3 axes were shown to protect against pressure-overload-induced cardiac dysfunction through regulation of lymphangiogenesis (Lin et al., 2021), Also, CU06-1004 was shown to enhance vascular integrity and improve cardiac remodeling by suppressing edema and inflammation upon myocardial ischemia-reperfusion injury (Zhang H. et al., 2022).

Notably, exaggerated left ventricular wall edema observed in Protein Kinase AMP-Activated Catalytic Subunit Alpha 1 deficient mice was shown not to be coupled with aggravated systolic dysfunction (decreased EF and FS) upon sepsis. However, it is suggested to contribute to diastolic dysfunction since end diastolic LV volume was significantly diminished in AMPK deficient mice (Castanares-Zapatero et al., 2013). Consistently, endothelial specific disruption of FLT1 in ECs (which increased KDR signaling) combined with administration of VEGFB-expressing AAV increased cardiac capillary density and leakage. This was associated with cardiac hypertrophy but not systolic dysfunction (Kivelä et al., 2019). Diastolic function was not assessed. Very recently, cardiac edema observed in mice overexpressing PDZ Domain Containing Ring Finger 3 (PDZRN3) specifically in ECs was shown to be sufficient to induce diastolic dysfunction (increased EDP and dP/dt min, increase NPPA and NPPB levels) without any signs of inflammation and fibrosis (Abelanet et al., 2022).

##### Fibrosis

Tissue fibrosis is characterized by a pathological accumulation of extracellular matrix (ECM) due to sustained activation of fibroblasts, referred to as myofibroblasts. This pathological accumulation of ECM increases tissue stiffness and may then be responsible for cardiac wall stiffening.

ECs may promote cardiac fibrosis through the production of pro-fibrotic molecules, notably TGFβ1, CTGF, and EDN1 (Sun et al., 2020a). EC specific disruption of EDN1 was shown to prevent hyperglycemia-induced capillary rarefaction, CDH5 downregulation, TGFβ1 upregulation and cardiac fibrosis in mice (Widyantoro et al., 2010). Also, TGF-β1-containing exosomes from cardiac microvascular ECs were shown to promote cardiac fibroblast activation under high glucose conditions (Zhang Y. et al., 2021) both *in vitro* and *in vivo*.

Alternatively, cardiac fibrosis was proposed to be favored by EC activation, which subsequently led to inflammatory cells infiltration in the heart. Both macrophage-derived Interleukin-10 (IL-10) (Zhang N. et al., 2022) and T-cell derived Interleukin-18 (IL-18) (Yu et al., 2009) were shown to promote cardiac fibroblast activation. Besides, lymphocytes were shown to be necessary for cardiac fibrosis and diastolic dysfunction (increased EDP) in HFD-fed mice using SCID mice (Zibadi et al., 2010).

Finally, ECs may directly contribute to fibrosis via phenotypic transition of ECs into mesenchymal cells, referred to as endothelial to mesenchymal transition (EndoMT). EndoMT may by induced by Transforming Growth Factor Beta 1 (TGFβ1), EDN1, hypoxia and disturbed blood flow (Sun et al., 2020a). However, even though EndoMT was first suggested to contribute significantly to cardiac fibrosis in the setting of pressure overload-induced cardiac hypertrophy using Tie1-Cre; Rosa26R mice (Zeisberg et al., 2007), later on EndoMT in the heart was shown to occur exclusively during development (Moore-Morris et al., 2014) by using an inducible promoter (notably in Cdh5-Cre/ERT2, RosatdT mice).

##### Inflammation

Inflammation is an important pathophysiological factor in both acute and chronic HF, predicting poor prognosis independently of LVEF (Murphy et al., 2020). Activated ECs express leucocyte adhesion molecules such as SELE, ICAM-1, and Vascular Cell Adhesion Molecule 1 (VCAM-1) which allows leucocytes to roll, adhere and transmigrate through ECs into the tissues. Besides, activated ECs synthesize cytokines, including interleukin 6 (IL-6), which regulates the acute phase response, and chemoattractants such as C-X-C Motif Chemokine Ligand 8 (CXCL8) and C-C Motif Chemokine Ligand 2 (CCL2). Also, expression of class II human leucocyte antigen (HLA) molecules allows ECs to act as antigen-presenting cells (Hunt and Jurd, 1998). Accordingly, disruption of *Icam-1* was shown to prevent TAC-induced leucocyte recruitment and cardiac fibrosis. Importantly, decreased leucocyte recruitment and fibrosis observed in Icam-1 deficient mice undergoing TAC were associated with a significant amelioration of diastolic (decreased EDP, increased dP/dt min) and systolic function (increased FS and dP/dT max) (Salvador et al., 2016). Decreased fibrosis and improved cardiac function observed in these mice was attributed to the diminished T-cell cardiac infiltration as TCRα deficient mice display a similar phenotype (Nevers et al., 2015).

Macrophages *via* Il-10 production (Zhang N. et al., 2022) and neutrophils via neutrophil extracellular traps (NETs) (Zhang X.-L. et al., 2022) were shown to promote diastolic dysfunction (increased EDP and Tau) in a mouse model of hypertension induced by salty drinking water, unilateral nephrectomy, and chronic exposure to aldosterone. We have shown that mast cells via histamine release promote diastolic dysfunction (increased EDP) in Lerp^db/db^ mice (Guimbal et al., 2021).

However, inflammatory cells do not always have detrimental effect. A recent study demonstrated that depletion of resident macrophages with a monoclonal α-CD115 antibody did not affect TAC-induced cardiac hypertrophy but did exacerbate TAC-induced cardiac fibrosis and systolic dysfunction. Depletion of CCR2+ macrophages recruited from the bone marrow prevented cardiac fibrosis demonstrating that bone marrow-derived macrophages, on the contrary to resident ones are detrimental (Revelo et al., 2021).

Inflammatory cytokines were shown to activate cardiac fibroblasts (see in the above paragraph), to modify cardiomyocyte phenotype and to exacerbate endothelial dysfunction. Notably, Tumor necrosis Factor (TNF) infusion for 15 days was shown to induce systolic dysfunction (decreased FS) in rats (Bozkurt et al., 1998). The same results were obtained with IL-1β in mice (Van Tassell et al., 2013). In both cases cardiac dysfunction was shown to be reversible. At cellular level, TNF induced negative inotropic effect and promoted apoptosis of cardiomyocytes (Bozkurt et al., 1998). IL-1β was shown to reduce beta-adrenergic responsiveness of L-type calcium channels and the expression of genes involved in the regulation of calcium homeostasis and to stimulating apoptosis in cardiomyocytes (Szekely and Arbel, 2018).

Altogether these results demonstrate that EC activation, by promoting inflammatory cells recruitment may participate in the pathophysiology of HF since many inflammatory chemokines were shown to have detrimental effects on cardiac function. Notably, neither macrophages *via* Il-10 nor mast cells *via* histamine did participate in the development of cardiac hypertrophy (Guimbal et al., 2021; Zhang N. et al., 2022).

However, it is not known whether inflammation is upstream or downstream of endothelial activation.

#### Conclusion

**FIGURE 3:**
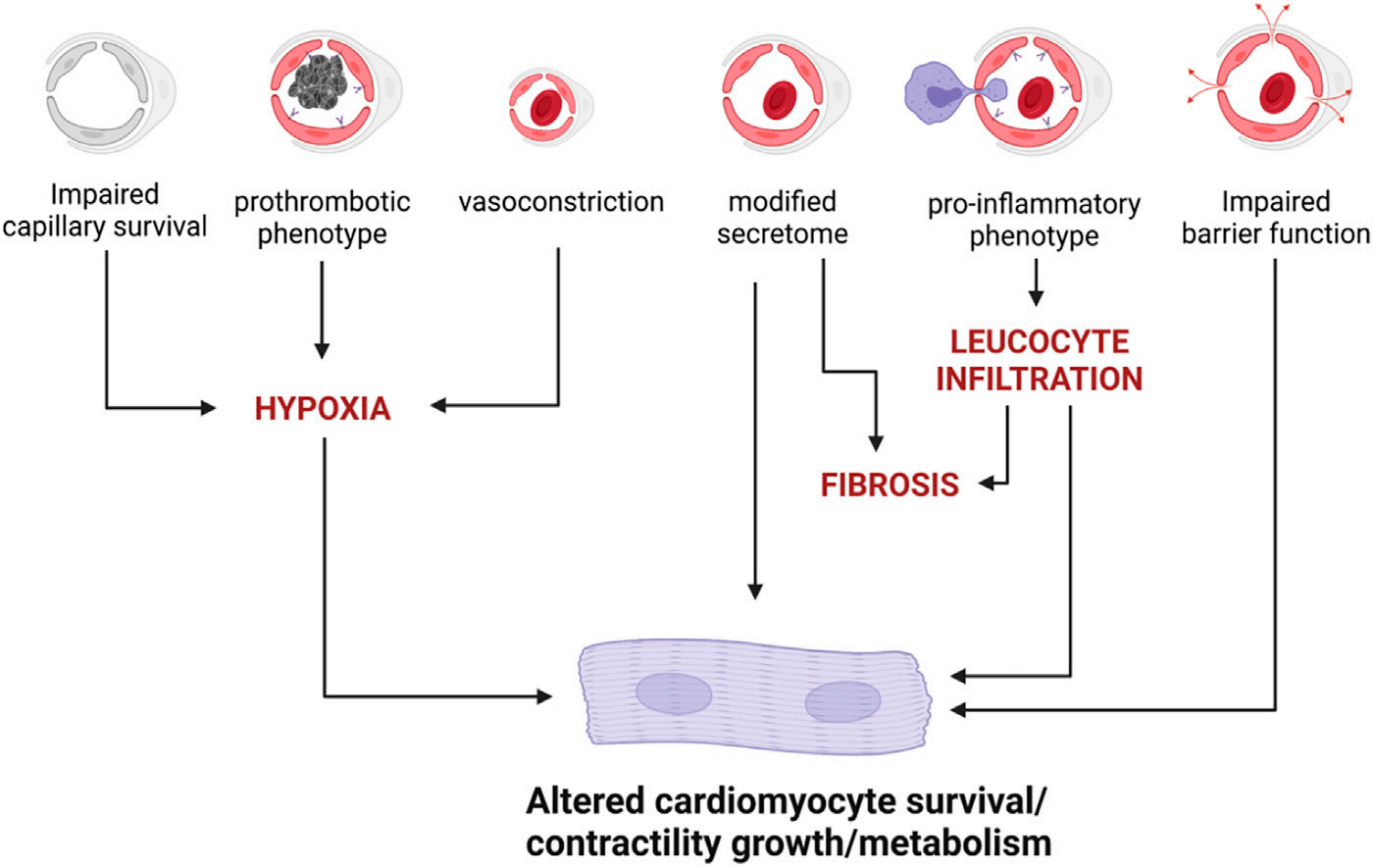
EC dysfunction may induce cardiomyocyte impairment *via* several mechanisms.

Altogether these data demonstrate that ECs may indeed affect cardiomyocyte homeostasis and function in many ways (Figure 3). However, it also highlights that some paradigms are far from being fully understood and demonstrated. Notably, none of the proposed mediators of the endothelial to cardiomyocyte crosstalk i.e., NO, NRG1, APLN or EDN1 seems to exert its action as a molecule released by ECs and acting on cardiomyocytes. On the contrary, they all seem to act autocrinally.

Besides, the fact that impaired NOS3 activity in ECs may actually be responsible for decreased Titin N2B phosphorylation and cardiomyocyte hypertrophy, as proposed by Paulus and Tschope (Paulus and Tschöpe, 2013), has not been demonstrated.

In summary whether or not endothelial dysfunction may participate in the pathophysiology of HF, especially HFpEF remains to be demonstrated.

### Genetic Modification of EC Properties is Sufficient to Cause Cardiac Dysfunction: Experimental Proofs

In order to demonstrate the specific consequence of endothelial dysfunction on cardiomyocytes and heart function, several groups have actually assessed the cardiac phenotype of mice with endothelial specific KO. The results of these studies are summarized below and may give clues and new insights regarding EC-cardiomyocyte crosstalk.

#### Genetic Modification of EC Properties Is Sufficient to Cause HF

Studies have demonstrated that genetic modification of endothelial properties, alone is sufficient to cause HF; this is the case of Platelet and EC Adhesion Molecule 1 (*Pecam1*) disruption, constitutive activation of Catenin Beta 1 (CTNNB1), Sirtuin 3 (*Sirt3*) disruption, Sirtuin 1 (*Sirt1*) disruption, Recombination Signal Binding Protein for Immunoglobulin kappa J region (*Rbpj*) deletion, EPH Receptor B4 (*EphB4*) deletion, β3-Adrenergic Receptor (ADRB3) overexpression and NOX2 overexpression (Table 1).

**TABLE 1:** Genetic modifications of EC sufficient to induce cardiac dysfunction.

Genetic Modification	Age/sex of mice	Mechanism	Modifications of cardiac properties	Type of dysfunction
*Sirt3* ECKO He et al. (2017)	12 month-old male mice	↘ Capillary density	↘ CFR	Diastolic dysfunction
		↘ glycolysis	↗ IVRT	
		↗ ROS	↗ MPI	
*Sirt3* ECKO Zeng et al. (2020b)	10 week-old female mice		↘ E'/A′	Diastolic dysfunction
			↗ IVRT	
			↗ MPI	
*Sirt1* ECKO Maizel et al. (2014)	30–40 week old	↘ Capillary density	↗ IVRT	Diastolic dysfunction
		↔ HIF1a	↗ MPI	
		↔ Fibrosis	↘ ATP2A2	
EC Tg (ADRB3) Dhot et al. (2020)	45 week-old male rats	↗ ROS (O2-)	↗ E/A	Diastolic dysfunction
		↗ NO	↗ Left atrial dilatation	
		↘ NOS3	↗ EDP	
		↗ NOS1 & NOS2	↘ Contractility (under stress)	
EC Tg (PDZRN3) Abelanet et al. (2022)	12 week old mice (8 weeks post-TAM)	↗ permeability	↗EDP	Diastolic dysfunction
		↔ capillary density	↗NPPA, NPPB	
		↔ inflammation	↗ dP/dt min	
		↔ Fibrosis		
*Rbpj* ECKO Jabs et al. (2018)	12 to 18 week-old mice (6–8 weeks post-TAM)	↘ FA uptake	↗ Hypertrophy	Systolic dysfunction
		↘ LIPG	↗ CM size	
		↗ Glucose uptake	↘ EF	
		↗ ANGPTL4	↘ FS	
*Pecam1* KO McCormick et al. (2015)	12 to 16-week-old mice	↗ NO	↘ EF	Systolic dysfunction
		↗ ROS	↘ FS	
		↗ NRG1	↗ Hypertrophy	
EC tg (CTNNB1∆E4) Constitutively active mutant Nakagawa et al. (2016)	8 to 10 week-old male (1 week post TAM)	↔ Capillary density	↗ Hypertrophy	Systolic dysfunction
		No hypoxia	↘ Survival at 50 weeks	
		↗ Fibrosis (COL1A1, COL3A1)	↘ FS	
		↘ NRG1	↗NPPA, NPPB	
*Ephb4* ECKO Luxán et al. (2019)	12 week-old mice (4 weeks post TAM)	↘ Vascular density	↗ hypertrophy	Systolic dysfunction (dilated cardiomyopathy)
		↘ Coronary vessel branch points	↗ CM size	
		↘ Pericytes	↘ EF	
		↗ Permeability to RBC		
*Tg(FOXO1-CA)* Dharaneeswaran et al. (2014)		↔ Capillary density	↘ cardiac output	Cardiac dilation
		↘ Capillary lumen (endothelial hypertrophy)		

Sirt3, Sirtuin 3; ECKO, endothelial cell specific knock out; Sirt1, Sirtuin 1; ADRB3, Adrenoceptor Beta 3; tg, transgenic; PDZRN3, PDZ Domain Containing Ring Finger 3; Rbpj, Recombination Signal Binding Protein For Immunoglobulin Kappa J Region; Pecam1, Platelet And Endothelial Cell Adhesion Molecule 1; CTNNB1, Catenin Beta 1; Ephb4:, EPH Receptor B4; FOXO1, Forkhead Box O1; ROS, reactive oxygen species; RBC, red blood cells; EF, ejection fraction; TAM, tamoxifen; CM, cardiomyocyte; EDP, end diastolic pressure; CFR, coronary flow reserve; IVRT, isovolumic relaxation time; MPI, myocardial performance index; FA, fatty acid.

##### Genetic Modification of EC Properties May Cause Systolic Dysfunction

Notably, *Pecam1* disruption (*Pecam1* constitutive KO) (McCormick et al., 2015), constitutive activation of CTNNB1 (CTNNB1 deleted from its degradation domain) (Nakagawa et al., 2016), *Rbpj* disruption (Jabs et al., 2018) and *EphB4* disruption (Luxán et al., 2019) were shown to induce systolic dysfunction characterized by increased Natriuretic Peptide A (NPPA) or Natriuretic Peptide B (NPPB), decreased EF, decreased FS and cardiac hypertrophy expect for *EphB4* disruption which did induce cardiomyocyte hypertrophy but not cardiac hypertrophy. While cardiomyocyte contractility was shown to be severely altered in mice with constitutive activation of CTNNB1 (dilated T-tubules and degenerated mitochondria), cardiomyocyte contractility measured *ex vivo* was normal in Pecam1 deficient mice. Besides, neither Pecam1 disruption nor CTNNB1 constitutive activation modified capillary density; on the contrary *Rbpj* ^ECKO^ increased cardiac capillary density while *EphB4* ^ECKO^ reduced cardiac capillary branching. Moreover, in *EphB4* ^ECKO^ mice, the barrier properties of ECs were compromised with impaired CDH5 dependent junction and capillary leakage evidenced by red blood extravasation, this was associated with a significant pericyte loss.

In the first two models, the “cardiac phenotype is proposed to be due to altered NRG1 expression levels by ECs, nevertheless, while *Pecam1* KO was associated with increased NRG1 levels and increased NRG1-induced signaling (ERBB2 phosphorylation), CTNNB1 constitutive activation was associated with decreased NRG1 levels and decreased NRG1-induced signaling (ERBB2 and ERBB4 signaling phosphorylation). In both models the authors have performed rescue experiments by either administrating NRG1 blocking antibodies in *Pecam1* deficient mice of NGR1 recombinant protein in mice expressing active CTNNB1 and observed a phenotype correction. However, endothelium specific *Nrg1* knockout mice were shown to display no significant difference in wall thickness, left ventricular chamber size, or systolic function (Hedhli et al., 2011) suggesting that NRG1 downregulation might not be responsible for the systolic dysfunction observed in mice with CTNNB1 constitutive activation in ECs (Luxán et al., 2019).

In both *Rbpj* ^ECKO^ and *EphB4* ^ECKO^ mice, systolic dysfunction was associated with decreased fatty acid uptake. The phenotype was further explored in *Rbpj* ^ECKO^ mice in which decreased fatty acid uptake was associated with increased glucose uptake leading to cardiac accumulation of Glucose-6-Phosphate subsequently leading to sustained AKT1 and Mechanistic Target Of Rapamycin Kinase (MTOR)- Ribosomal Protein S6 Kinase (RPS6K) signaling. Notably, Notch inhibition in ECs was shown to induce decreased transcription of endothelial lipase (LIPG) which hydrolyzes mainly phospholipids but also triacylglycerol, increases expression of ANGPTL4 (noncompetitive lipoprotein lipase inhibitor angiopoietin-like 4 (ANGPTL4)), and decreases expression of CD36, and Fatty Acid Binding Protein 4/5 (FABP4/5*)* (Jabs et al., 2018)*.* Consistently, *Lipg* deficient were shown to display exacerbated cardiac hypertrophy and HF upon TAC (Nakajima et al., 2013) and *Fabp4/5* double KO mice were shown to display cardiac hypertrophy (Iso et al., 2013).

None of these mice were shown to display increased cardiac fibrosis.

##### Genetic Modification of EC Properties May Cause Diastolic Dysfunction

On the contrary, *Sirt3* or *Sirt1* disruption and ADRB3, NOX2 and PDZRN3 overexpression in ECs were shown to induce diastolic dysfunction. More specifically, *Sirt3* (He et al., 2017) and *Sirt1* endothelial specific disruption was shown to increase IVRT and/or E/e’ ratio (Maizel et al., 2014). This was associated with diminished ATP2A2 levels in *Sirt1* deficient mice. While *Sirt3* ^ECKO^ mice display increased perivascular fibrosis, *Sirt1* ^ECKO^ mice did not. Besides, both *Sirt3* ^ECKO^ and *Sirt1* ^ECKO^ mice have decreased cardiac capillary density and decreased CFR. Mechanistically, *in vitro* experiments showed that deletion of endothelial *Sirt3* impairs glycolytic activity, increased oxygen consumption and ROS production in ECs. Notably, the phenotype was proposed to be more severe in female since *Sirt3* ^ECKO^ female mice develop cardiac hypertrophy while *Sirt3* ^ECKO^ male mice do not (He et al., 2017; Zeng et al., 2020b). In rats overexpressing ADRB3, diastolic dysfunction was evidenced by increased EDP and increased E/A ratio (Dhot et al., 2020). In these rats, diastolic dysfunction was associated with increased ROS production and cardiac fibrosis but not cardiac hypertrophy. Capillary density was unchanged so was cardiac inflammation. On the contrary to *Sirt3* ^ECKO^ mice, only male overexpressing ADRB3 developed diastolic dysfunction (Dhot et al., 2021). Finally, in mice overexpressing PDZRN3 diastolic dysfunction was evidenced by increased EDP and dP/dt min and increased NPPA and NPPB levels. In these mice, diastolic dysfunction was associated with cardiac edema but not cardiac inflammation, fibrosis or hypertrophy (Abelanet et al., 2022).

Notably, except for *Sirt3* ^ECKO^ female mice, in none of these mice, diastolic dysfunction was associated with cardiac hypertrophy.

#### Genetic Modification of EC Properties May Exacerbate HF

Other studies have revealed that some other genetic modification of endothelial properties exacerbates HF induced by ANG2 infusion or TAC while they are not sufficient to induce HF by themselves. This is the case of Forkhead Box P1 (*Foxp1*), Sphingosine-1-Phosphate Receptor 1 (*S1pr1*) or Hypoxia Inducible Factor 1 Subunit Alpha (*Hif1a*) disruption and NOX2 overexpression (Table 2).

**TABLE 2:** Genetic modifications of EC exacerbating cardiac dysfunction.

Genetic Modification	Model	Mechanisms	Modifications of cardiac properties	Type of dysfunction
*Lipg* KO Nakajima et al. (2013)	TAC 12-week-old male mice	↘ β-oxidation	↗ Hypertrophy	Systolic dysfunction
			↘ FS	
			↗ NPPA and NPPB	
			Pulmonary edema	
EC Tg (NOX2) Murdoch et al. (2014)	ANG2	↗ ROS	↘ LV end-systolic volume	Diastolic dysfunction
	1.1 mg/kg/day	↗Fibrosis (COL1A1)	↘ Stroke volume	
	14 days	↗Inflammation (CD45^+^, Mac3+)	↘ LV end-diastolic dimension and volume	
	12-week-old male mice			
S*1pr*1 ECKO Liu et al. (2020)	TAC (1 week after TAM injection)	↗ Fibrosis	↗Hypertrophy	Systolic dysfunction
			↘ FS	
			↘ EF	
*Foxp1* ECKO Liu et al. (2019)	ANG2	↗ Fibrosis (COL1A1, COL3A1, TGFβ 1, fibroblast proliferation)	↗ Hypertrophy	Diastolic dysfunction
	1.44 mg/kg/day		↗E/e'	
	14 days		↗CM size	
	(1 week after TAM injection)			
*Hif1a* ECKO Wei et al. (2012)	TAC male mice	↘ Vascular density	↘ EF	Systolic dysfunction
		↗ Myocardial hypoxia	↘ FS	
		↗ Fibrosis (TGF-β)	↗ Hypertrophy	
*Sirt3* ECKO Zeng et al. (2020b)	TAC 7 weeks after	↘ APLN	↗ Hypertrophy	Systolic dysfunction
		↘ HIF1a	↘ EF	
		↘ GLUT1	↘ FS	
		↘ Glucose uptake		

Lipg, Lipase G; Endothelial Type, NOX2, NADPH Oxidase 2; S1pr1, Sphingosine-1-Phosphate Receptor 1; Foxp1, Forkhead Box P1; Hif1a, Hypoxia Inducible Factor 1 Subunit Alpha; Sirt3, Sirtuin 3; TAC, transverse aortic constriction; TAM, tamoxifen; ANG2, Angiotensin 2; ROS, reactive oxygen species; FS, fractional shortening; EF, ejection fraction; LV, left ventricular.

More specifically, both loss of endothelial *Foxp1* (Liu et al., 2019, 1) or NOX2 overexpression (Murdoch et al., 2014) were shown to exacerbated ANG2-induced diastolic dysfunction (increased E/e’ or decreased end diastolic volume, increased LV diastolic stiffness and EDP) (Liu et al., 2019, 1). While endothelial deletion of *S1pr1* (Liu et al., 2020) or *Hif1a* (Wei et al., 2012) was shown to exacerbate TAC-induced cardiac hypertrophy and systolic dysfunction (decreased EF and FS). Notably, *Foxp1* ^ECKO^ did exacerbate ANG2-induced cardiac hypertrophy while NOX2 overexpression did not.

In each case, exacerbation of HF was associated with increased myofibroblast formation and subsequent cardiac fibrosis. Mechanistically, both *Foxp1* and *Hif1a* were proposed to limit fibrosis by downregulating TGFβ1 in ECs while *S1pr1* downstream signaling in ECs is proposed to promote eNOS phosphorylation via AKT1 leading to increased sGC activity in fibroblasts. Importantly, TGFβ1 blockade prevented HF exacerbation in both *Foxp1* ^ECKO^ and *Hif1a* ^ECKO^ mice (Wei et al., 2012; Liu et al., 2019, 1).

In addition, exacerbation of HF in *Foxp1* ^ECKO^ and *Hif1a* ^ECKO^ was associated with capillary rarefaction increasing cardiac hypoxia (Wei et al., 2012; Liu et al., 2019, 1). On the contrary, *S1pr1* ^ECKO^ and NOX2 overexpression did not induce capillary rarefaction, however, conditioned medium from S1PR1 overexpressing EC was shown to decrease MYH7, NPPA and NPPB in cardiomyocytes, suggesting that *S1pr1* deletion in ECs may also modify cardiomyocyte phenotype independently on cardiac fibrosis (Liu et al., 2020). Tg (NOX2) mice display significant cardiac infiltration of leucocytes associated with increased VCAM-1 levels (Murdoch et al., 2014).

*Sirt3* ^ECKO^ was shown to induce diastolic dysfunction alone (He et al., 2017; Zeng et al., 2020b), however, while associated with TAC, it was actually shown to exacerbate systolic dysfunction (decreased EF and FS) and TAC-induced cardiac hypertrophy (Zeng et al., 2020a). Mechanistically, Sirt3 disruption in ECs is proposed to prevent hypoxia-induced APLN overexpression and subsequently glucose uptake in both ECs and cardiomyocytes via the regulation of Solute Carrier Family 2 Member 1 (SLC2A1) and/or Solute Carrier Family 2 Member 4 (SLC2A4) expression.

#### Summary

**FIGURE 4:**
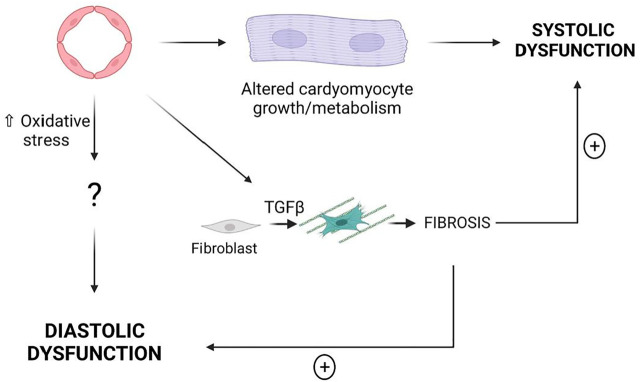
EC dysfunction may induce either systolic dysfunction or diastolic dysfunction. Cardiac Fibrosis is not sufficient to induce HF however it may exacerbate both systolic dysfunction and diastolic dysfunction.

Taken together these results demonstrate that modifications of EC properties are sufficient to induce cardiac dysfunction, notably some of these modifications induce systolic dysfunction while some other induce diastolic dysfunction. Endothelial-induced systolic dysfunction is associated with cardiac hypertrophy while endothelial-induced diastolic dysfunction is associated with oxidative stress (Figure 4).

Whether or not these mechanisms may actually participate in HFpEF pathophysiology remain to be verified. Indeed, Pecam1 disruption, constitutive activation of CTNNB1, *Rbpj* disruption and *EphB4* disruption which induce systolic dysfunction are more likely not involved in HFpEF. On the contrary, NOX2 overexpression or SIRT1 downregulation may participate in the pathophysiology of HFpEF since NOX2 overexpression in ECs induce diastolic dysfunction and NOX2 was shown to be upregulated in HFpEF patients (Franssen et al., 2016). Similarly, low activity of SIRT1 in peripheral blood mononuclear cells have been specifically associated with HFpEF but not HFrEF or HF with mid rang EF (HFmrEF) (Conti et al., 2020) and *Sirt1* ^ECKO^ was shown to induce diastolic dysfunction.

Modulation of pro-fibrotic signaling pathways (i.e., increased TGFβ1 expression upon *Foxp1* or *S1pr1* KO) does not seem to be sufficient to induce HF, on the contrary it may exacerbate HF in combination with other factors.

Finally, these studies identify a new critical role of cardiac ECs in regulating cardiomyocyte metabolism.

### EC Dysfunction in Models of “HFpEF”

Investigating the role of EC dysfunction in HFpEF pathophysiology in experimental models or in humans has been difficult because HFpEF is a multifactorial heterogeneous disease and because a single animal model recapitulating most clinical features of this disease does not exist (Withaar et al., 2021a). Currently used animal models of HFpEF recapitulate one or several risk factors for HFpEF and display an association of HFpEF clinical features but do not meet the entire heterogeneity of the clinical HFpEF syndrome. Most of them present a diastolic dysfunction with an elevation of EDP and a preserved EF. The models recapitulating a combination of cardiovascular risk factor or “multi-hit models” are considered the best ones (Withaar et al., 2021a).

Even though HFpEF have been shown strongly to be associated with endothelial dysfunction in human studies, the following paragraph will highlight that the phenotype of the cardiac vasculature has not been extensively described in animal models (Table 3).

**TABLE 3:** Microvascular characteristics of rodent models of HFpEF.

	Capillary density	Vasomotricity	NO/sGC-GMPc-PRKG1	Oxidative stress	Endothelial activation	Permeability	Pericyte coverage	Glycocalyx	Fibrosis	Inflammation
One hit models										
*Lepr* ^db/db^ mice	↘	ND	↘	ND	↗	↗	ND	↘	=/↗	↗
HFD/WD mice	ND	ND	ND	↗	ND	ND	ND	ND	↗	ND
Aged mice	↘	ND	ND	ND	ND	ND	ND	ND	↗	ND
SAMP8 mice	ND	↘	↘	ND	↗	ND	ND	ND	↗	
DOCA-salt mice	↘	↘	↘	↗	ND	ND	ND	ND	=	ND
Aldosterone uninephrectomy mice	ND	ND	↘	↗	↗	ND	ND	ND	↗	↗
Dahl salt Sensitive Rats	=	↘	↘	↗	↗	ND	ND	ND	↗	↗
ANG2 infusion mice	ND	ND	ND	ND	ND	ND	ND	ND	↗	ND
Two hit models										
ZSF1 rats	ND	↘	↘	↗	↗	ND	ND	ND	↗	↗
HFD + L-NAME mice	↘	↘	↘	ND	ND	ND	ND	ND	↗	ND
HFD + ANG2 mice	ND	ND	ND	↗	ND	ND	ND	ND	↗	↗
SAMP8+WD	ND	↘	↘	ND	↗	ND	ND	ND	ND	ND
Three hit models										
Age + HFD + ANG2	↘	ND	ND	ND	ND	ND	ND	ND	↗	↗
Age + HFD + DOCA	ND	↘	ND	↗	ND	ND	ND	ND	↗	↗

Lepr, Leptin receptor; HFD, high fat diet; WD, western diet; SAMP8, senescence accelerated mouse prone 8; DOCA, Deoxycorticosterone acetate; ANG2, Angiotensin 2; ZSF1, Zucker fatty/Spontaneously hypertensive heart failure F1 hybrid; L-NAME, N(ω)-nitro-L-arginine methyl ester; ND, not determined.

#### Rodent Models

##### One Hit Models

###### Mouse/Rat Models With Metabolic Disease

Both male and female leptin receptor deficient *Lepr* ^db/db^ mice develop severe obesity and hyperglycaemia; females have more impressive weight gain and exhibit an increased blood pressure.

Consistent with HFpEF, these mice display signs of diastolic dysfunction (increased EDP, E/A and E/e’ as soon as 3 months of age) with a preserved EF. This is associated with hypertrophic ventricular remodeling and pulmonary edema. Whether or not these mice display cardiac fibrosis is controversial (Alex et al., 2018; Guimbal et al., 2021). This model is actually the one in which the cardiac vasculature has been the best characterized. Notably, these mice were shown to display rarefaction of cardiac capillaries, endothelial activation (increased ICAM-1, SELE), and capillary leakage (Guimbal et al., 2021). Besides, these mice were shown to have a decreased endothelial glycocalyx (Qiu et al., 2022) and impaired peripheral vasorelaxation to carbamoylcholine (Otto et al., 2021). Finally, the phenotype of cardiac ECs have been completed by a transcriptomic analysis via RNA sequencing (Guimbal et al., 2021).

The endothelial phenotype has not been characterized in mouse models in which metabolic disease was induced by high fat (HFD) or western diets. HFD was shown to induce diastolic dysfunction characterized by decreased dP/dt max and dP/dt min and associated with cardiac hypertrophy, fibrosis and inflammation (Aroor et al., 2017; Bostick et al., 2017; Ternacle et al., 2017).

###### Mouse Models of Aging

Since HFpEF is a disease associated with aging both SAMP8 (Senescence accelerated prone mice) (Gevaert et al., 2017) and 24-30-month-old mice (Roh et al., 2019) have been used as models of HFpEF. Accordingly these mice were shown to display diastolic dysfunction (decreased of E/A in SMAP8 mice, increased BNP in aged mice) with preserved EF and cardiac remodeling including LV hypertrophy and fibrosis (Reed et al., 2011). SAMP8 mice display exercise intolerance and lung edema. At vascular level, SAMP8 were shown to display impaired acetylcholine-dependent relaxation associated with decreased of NOS3 levels, endothelial activation (increased ICAM-1) and senescence (Gevaert et al., 2017) while 24-30-month-old mice were shown to have a cardiac capillary rarefaction.

###### Rat/Mouse Models of Hypertension

Rodent models of hypertension used as mouse models of HFpEF, include mice fed with high-salt diet and treated with deoxycorticosterone acetate (DOCA) (DOCA-salt), mice infused with ANG2, mice with aldosterone infusion and uninephrectomy and Dahl salt-sensitive rats.

In DOCA-salt mice, diastolic dysfunction has been evidenced by an increased EDP, a decreased E’/A′ and an increased E’/e and associated with cardiac hypertrophy but not cardiac fibrosis (Mohammed et al., 2010; Silberman et al., 2010; Lovelock et al., 2012; Bowen et al., 2017).

At the vascular level, these mice were shown to have impaired NOS3 activity with decreased NO production and increased ROS production (Silberman et al., 2010) associated with increased expression of NOX2, NOX4 and Cytochrome B-245 Alpha Chain (CYBA) (Selma F. Mohammed et al., 2010).

Mice that underwent uninephrectomy and aldosterone infusion display diastolic dysfunction characterized by decreased E/A and increased IVRT and decreased deceleration time, lung edema and increased NPPA and NPPB (Valero-Munoz et al., 2016). This diastolic dysfunction was accompanied by cardiac hypertrophy and fibrosis (Wilson et al., 2009; Valero-Munoz et al., 2016; Zhang L. et al., 2021). At the endothelial level, these mice were shown to have decreased phosphorylated NOS3 levels and endothelial activation characterized by increased soluble VCAM-1 levels (Wilson et al., 2009; Zhang L. et al., 2021).

Dahl salt-sensitive rats fed with high salt diet have diastolic dysfunction (increased EDP), characterized by impaired cardiac relaxation (decreased dP/dt min and increased Tau) (Adams et al., 2015; Esposito et al., 2017) and associated with cardiac hypertrophy, fibrosis and inflammation (Esposito et al., 2017). In these rats, endothelial dysfunction was also characterized by endothelial activation (increased VCAM-1 and SELE) and NOS3 uncoupling leading to decreased NO production, impaired endothelium-dependent and vasodilatation to Ach and increased oxidative stress (increased NOX2) (Adams et al., 2015, 1).

The vascular phenotype was not assessed in mice infused with either ANG2 (Ichihara et al., 2001; Regan et al., 2015) in which diastolic dysfunction has been characterized by increased EDP, IVRT and Tau and a lower E/A ratio. Diastolic dysfunction was associated with cardiac hypertrophy and fibrosis in these models.

##### Two Hit Models

Diabetic, obese and hypertensive ZSF1 rats are obtained by breeding leptin resistant Zucker Diabetic Fatty (ZDF) with spontaneously hypertensive Heart Failure (SHHR) rats. These rats develop diastolic dysfunction with elevation of EDP and impaired cardiac relaxation (diminished dP/dt min) and that is associated with cardiac hypertrophy, fibrosis and inflammation. (Franssen et al., 2016; Schmederer et al., 2018; Leite et al., 2019; Schauer et al., 2021). At vascular level, ZSF1 were shown to display endothelial activation (increased ICAM-1 and SELE), decreased NOS3 phosphorylation and coupling which is associated with impaired endothelial-mediated vasodilatation to acetylcholine and decreased artery compliance.

In mice, combination of metabolic disease with hypertension was achieved either by combining and HFD regimen with L-NAME or with ANG2 treatment.

In HFD+L-NAME-treated mice, diastolic dysfunction was characterized by increased EDP and was associated with cardiac hypertrophy and fibrosis. The vascular dysfunction was evidenced by capillary rarefaction (Schiattarella et al., 2019). Consistent with the continuous treatment by L-NAME, these mice were shown to display impaired endothelial-dependent vasodilatation to acetylcholine (Cordero-Herrera et al., 2020).

In HFD+ANG2-treated mice, diastolic dysfunction was evidenced by increased E/A, diminished end diastolic volume and decreased dP/dt min and associated with cardiac hypertrophy, fibrosis, and inflammation (Du et al., 2018; Reddy et al., 2018). The cardiac vascular phenotype was not assessed in this model except that HIF1α was shown to be upregulated suggesting cardiac hypoxia.

One model combined aging with metabolic disease. Indeed, SAMP8 mice were fed with a western diet for 24 weeks and shown to display enhanced features of diastolic dysfunction with increased EDP, E/e’ and NPPB compared to SMAP8 mice on a show diet (Gevaert et al., 2017). This was associated with cardiac hypertrophy, fibrosis. At the vascular level, these mice were shown to have decreased NOS3 levels and impaired endothelium-dependent vasodilation, endothelial activation with increased ICAM-1 levels and endothelial senescence (increased p53).

##### Three Hit Models

More recently 3-hit models have been developed; these models combine metabolic disease and hypertension with aging.

Notably, 18 to 22-month-old female mice were treated with HFD+ANG2 (Withaar et al., 2021b). In these mice HF was evidenced by increased NPPA and lung congestion and was associated with cardiac hypertrophy and fibrosis. Besides these mice were shown to display capillary rarefaction, endothelial apoptosis (increased expression of Fas cell surface death receptor (FAS), Caspase 3) and vascular calcifications (increased expression of TNF receptor superfamily member 12a (TNFRSF12A) and osteprotegerin).

In another model, HFpEF was induced by a **13-months HFD regimen combined with DOCA** treatment (Deng et al., 2021). Consistent with HFpEF, these mice showed impaired cardiac relaxation with decreased dP/dt min and lung congestion. Beside diastolic dysfunction was associated with cardiac hypertrophy, fibrosis and inflammation. Assessment of the vascular phenotype was limited to the measure of endothelium-dependent vasorelaxation.

##### Large Animal Models

Large animal models of HFpEF also include single and multi-hit models (Table 2).

###### Hypertensive Dogs

Hypertension was induced in old dogs by renal wrapping and administered with desoxycorticosterone pivalate (DOCP) to exacerbate inflammation. These dogs display diastolic dysfunction characterized by increased EDP and decreased relation constant Tau and associated with cardiac hypertrophy and fibrosis (Munagala et al., 2005; Hamdani et al., 2013; Zakeri et al., 2016). At the vascular level, these dogs display both capillary rarefaction and vasoconstriction associated with impaired NO signaling (Zakeri et al., 2016).

###### Pigs With Hypertension and Hyperlipidaemia

Hypertension and hyperlipidemia were induced in pigs by a western diet (high salt, fat, cholesterol and carbohydrates) and DOCA administration. This induced diastolic dysfunction characterized by a shift leftward of the EDP-volume loop and increased EDP and E/e’. In this model diastolic dysfunction was associated with LV concentric hypertrophy, cardiac fibrosis (Schwarzl et al., 2015; Sharp et al., 2021) and impaired vasorelaxation to endothelial-dependent agonists, bradykinin and substance P (Sharp et al., 2021).

Alternatively, Pigs were fed with a western diet, administered with DOCA and infused with ANG2. In this model, diastolic dysfunction was characterized by increased IVRT, decreased dP/dt max and min and associated with cardiac hypertrophy, fibrosis and inflammation. In this model NO signaling was also shown to be impaired with a reduced expression of NOS3, PRKG1 and cGMP content in the aorta (Zhang et al., 2019).

###### Pigs With Hypertension, Hyperlipidaemia and Hyperglycaemia

In female pigs fed by an HFD regimen (hypercholesterolemia), administered with strepozotocin (hyperglycaemia) and subjected to renal artery embolism (hypertension), diastolic dysfunction was evidenced by elevated passive cardiomyocyte stiffness and increased left ventricular end-diastolic stiffness and associated with oxidative stress fibrosis and inflammation. The cardiac endothelial phenotype was characterized by a decreased capillary density, and an impaired endothelium-dependent vasodilatation to bradykinin because of NOS uncoupling and increased NOX activity (Sorop et al., 2018).

###### Pigs With Pressure Overload and Metabolic Disease

In this last model, diastolic dysfunction was induced by a western diet and a pressure overload caused by aortic banding and characterized by a shift in the EDP-volume curve. It was associated with cardiac hypertrophy, inflammation and impaired vasodilation to acetylcholine, insulin and sodium nitroprusside (Olver et al., 2019).

#### Summary

Characterization of endothelial dysfunction has been neglected in most animal models of HFpEF, Capillary density, endothelial dysfunction or oxidative stress have been assessed in only 6 of the 14-rodent models listed above (Tables 3, 4). Even NOS3 activity or endothelial-dependent vasodilation, the most studies feature of endothelial dysfunction has been evaluated in only 8 of the 14 models. Endothelial permeability which is suggested to be present in 66% of patients with HFpEF (Fischer et al., 2022) has been quantified in only one model.

**TABLE 4:** Microvascular characteristics of large HFpEF models.

	Capillary density	Endothelial activation	NO/sGC-GMPc-PRKG1	Vasomotricity	Oxidative stress	Permeability	Pericyte coverage	Fibrosis	Inflammation
Renal wrapping in dogs	↘	ND	↘	↘	ND	ND	ND	↗	ND
DOCA + WD in pigs	ND	ND	↘	↘	↗	ND	ND	↗	ND
HFD + renal artery embolization in pigs	↘	ND	↘	↘	ND	ND	ND	↗	↗
Stented pigs	ND	ND	ND	ND	ND	ND	ND	↗	ND
Aortic bending + WD in pigs	ND	ND	ND	↘	ND	ND	ND	↗	↗
DOCA + ANG2 + WD in pigs	ND	ND	↘	ND	ND	ND	ND	↗	↗

DOCA, Deoxycorticosterone acetate; WD, western diet; HFD, high fat diet; ANG2, Angiotensin 2; ND, not determined.

### Therapeutic Perspectives

Considering the critical role of the cardiac vasculature in providing oxygen, nutriments, signals and a “pleasant” environment for cardiomyocyte homeostasis and function, restoring endothelium integrity and function appears to be a valuable strategy to treat HF (Luxán and Dimmeler, 2021). However, to date, only empagliflozin, a Sodium-glucose cotransporter 2 (SGLT2) inhibitor (Anker et al., 2021) and preventive therapies targeting cardiovascular risk factors have been shown to reduce mortality and/or hospitalization in HFpEF patients. Even though, gliflozins were shown to improve endothelial dysfunction by preventing activation of ECs and accumulation of ROS, and by promoting AKT phosphorylation, NO production and vasodilation (effects all reviewed in (Alshnbari et al., 2020)). whether or not SGLT2 inhibitors improve cardiac function at least in part by improving endothelial dysfunction is not known (Pabel et al., 2021).

This paragraph will discuss the proposed mechanisms by which empagliflozin may improve HFpEF summarize the results of clinical trials testing the therapeutic potential of improving NO signaling and review the results of the preclinical studies in which authors have attempted to test whether improving endothelial dysfunction may treat HF.

#### Empaglyflozin

Empagliflozin, canagliflozin and dapagliflozin are sodium-glucose co-transporter 2 (SGLT2) inhibitors. This co-transporter is mainly expressed in the proximal tubular cells of kidneys. Gliflozins reduce renal reabsorption of glucose increasing its urinary excretion which decreases glycemia. They are indicated in type 2 diabetes and more recently in HF. Empagliflozin first demonstrated cardiovascular benefits in the EMPAREG-OUTCOME clinical trial (Sarafidis and Tsapas, 2016) and empagliflozin is the first drug that has shown reduction of hospitalizations of HFpEF patients in the EMPEROR-Preserved clinical trial (Anker et al., 2021).

Consistently, empagliflozin was shown to improve diastolic function by reducing LV end-diastolic volume, E/A and E/e’ ratios in patients (Cohen et al., 2019; Sakai and Miura, 2019). In animals, empagliflozin was shown to improve LV filling pressure (E/E’ ratio) in type 2 diabetic *Lepr* ^db/db^ mice (Habibi et al., 2017), and to improve cardiac relaxation (decreased Tau) and hypertrophy in DOCA salt non-diabetic rats (Connelly et al., 2019). However, the mechanism by which SGLT2 inhibitors improve cardiac function is unknown (Pabel et al., 2021).

First of all, empagliflozin may improve cardiac function by improving cardiovascular risk factors of HFpEF. Indeed, empagliflozin was shown to reduce systolic and diastolic blood pressure in patients with type 2 diabetes and HFpEF (Chilton et al., 2015; Sakai and Miura, 2019). Also, it was also shown to reduce triglycerides, cholesterol, glucose and HbA1c levels in both patients (Sakai and Miura, 2019) and animal models of diabetes and/or HFpEF (Connelly et al., 2019). Finally, by causing significant natriuresis, empagliflozin improves the blood volume (Griffin et al., 2020). Second, empagliflozin was shown to promote cardiomyocyte passive stiffness and to decrease cytosolic sodium and calcium levels *ex vivo* in isolated myocardial fibers possibly by enhancing phosphorylation of Titin and myofilament regulatory proteins (Pabel et al., 2018; Kolijn et al., 2021) and by inhibiting Na+/H+ 1 exchanger (NHE1) (Baartscheer et al., 2017) respectively. Third, empagliflozin may increase NO bioavailability. Notably, it was shown to promote NOS3 phosphorylation at Ser1177 (Oelze et al., 2014), and to increase sGC, PRKG1 and cGMP levels (Xue et al., 2019; Kolijn et al., 2021). Accordingly, empalgliflozin was shown to improve acetylcholine-induced endothelium-dependent vasodilation (Lin et al., 2014; Oelze et al., 2014; Aroor et al., 2018). Forth empagliflozin was shown to prevent accumulation of ROS in animal models of diabetes (Lin et al., 2014; Oelze et al., 2014; Kusaka et al., 2016), this has been associated with increased SOD2 and decreased NOX4 levels (Xue et al., 2019). Notably, empaglyflozin was shown to prevent ROS accumulation induced by TNF in human cardiac microvascular ECs and subsequently cardiomyocyte-impaired relaxation (Juni et al., 2019). Fifth, empagliflozin was shown to prevent cardiac fibrosis in diabetic *Lepr* ^db/db^ mice (Habibi et al., 2017; Xue et al., 2019), in pre-diabetic rats (Kusaka et al., 2016) and in non-diabetic rats (Byrne et al., 2020) possibly by downregulating TGFβ1 (Xue et al., 2019; Byrne et al., 2020). Diminished fibrosis was associated with decreased inflammation; notably CD68 macrophages were shown to be diminished so were several inflammatory cytokines including IL-6, CCL2 and TNF (Lin et al., 2014; Oelze et al., 2014; Kusaka et al., 2016). Additionally empagliflozin was shown to decrease the NLR Family Pyrin Domain Containing 3 (NLRP3) inflammasome which subsequently reduced Interleukin 1 beta (IL-1β) levels (Xue et al., 2019; Byrne et al., 2020). Finally, empagliflozin may protect ECs from apoptosis (Xue et al., 2019) and promote their quiescence (increased EC length) (Kolijn et al., 2021).

The beneficial effect of gliflozins in HFpEF has been confirmed in both the PRESERVED-HF and CHIEF-HF clinical trials in which dapagliflozin (Nassif et al., 2021) and canaglyflozin (Spertus et al., 2022) were shown the increased Kansas City Cardiomyopathy Questionnaire (KCCQ) Total Symptom Score and/or the 6-min walk test respectively.

#### Targeting NO

Following Paulus paradigm, several clinical trials have been designed to assess the therapeutic potential of restoring NO/sGC/cGMP signaling. However, in both the INDIE-HFpEF (Borlaug et al., 2018) and NEAT-HFpEF (Redfield et al., 2015) trials aiming at increasing NO bioavailability via administration of inhaled nebulized inorganic nitrite and isosorbide mononitrate respectively, the patients receiving the drug did not show significant improvement in exercise capacity compared to the one receiving the placebo. Three clinical trials have tested the potential benefits of stimulating sGC, in two of these trials, i.e., the VITALITY-HFpEF (Armstrong et al., 2020) and CAPACITY-HFpEF (Udelson et al., 2020), Vericiguat did not improve the physical limitation compared to placebo while praliciguat did not significantly improve peak V̇O2 from baseline. In the SOCRATES-Preserved trial, vericiguat did not change NT-proBNP and left atrial volume at 12 weeks compared with placebo but was associated with improvements in quality of life in patients with HFpEF (Pieske et al., 2017). Nevertheless, the combined results of these 3 trials together with the DILATE-1 trial did not demonstrate significant difference in 6-min walk test distance or the KCCQ physical limitation score in patients receiving sGC stimulator compared to patients that received placebo (Thakker et al., 2021). Finally, in the RELAX trail, aiming at increasing cGMP levels via phosphodiesterase-5 inhibition, HFpEF patients administration of sildenafil for 24 weeks, compared with a placebo, did not result in significant improvement in exercise capacity or clinical status (Redfield et al., 2013).

Altogether the negative results of these trials suggest that, on the contrary to the paradigm proposed by Paulus et al. (Paulus and Tschöpe, 2013), the NO/sGC/cGMP may not have a central role in the pathophysiology of HFpEF.

#### Targeting ECs

So far, evidence suggesting that improving EC dysfunction may improve HF exist only in preclinical studies. More specifically in the studies listed below, the authors have shown that endothelial specific KO/overexpression prevents in the occurrence of cardiac dysfunction-induced by hypertension, pressure-overload or metabolic diseases (Table 5).

**TABLE 5:** Genetic modifications of EC properties that protect from developing cardiac dysfunction.

	Model	Mechanism?	Cardiac phenotype	
*Nr3c2* ECKO Rickard et al. (2014)	DOC salt mice	↘ Inflammation (MΦ, CCR5, NOS2, PAI-1	ND	
		↘ Fibrosis (CTGF)		
		↔ PA		
*Nr3c2* ECKO Jia et al. (2015)	4-months HFD (45% kCal from fat) mice	↘ Fibrosis (TGFβ, COL1A1, FN)	↘ Diastolic relaxation time	↗ Diastolic function
		↘ Inflammation (M1 MΦ)	↘ IVRT	
		↘ ROS (peroxynitrite)	↗ E'/A	
Scnn1a ECKOSowers et al. (2020)	3-months HFD (45% kCal from fat) mice	↔ Fibrosis	↗ e’/a’	↗ Diastolic function
		↘ Oxidative stress	↘ E/e’	
		(NRF2)	↘ MPI	
			↘ CM stiffness	
			↔ Hypertrophy	
*Icam-1 KO* Salvador et al. (2016)	TAC mice	↘ Fibrosis	↘ Hypertrophy	↗ Diastolic and systolic function
		(TGFβ, COL1A1)	↘ EDP	
		↘ Inflammation	↘ dP/dt min	
		(CD45^+^, CD3^+^, CD4^+^, and Ly6G^high^)	↗ FS	
			↘ dP/dt max	
EC Tg (FOXP1) Liu et al. (2019), 1	ANG2 mice	↘ Fibrosis (SMA, COL1A1, COL3A1, VIM)	↘ Hypertrophy	↗ Diastolic function
		↔ PA	↘ E/e’	
*Ets1* ECKO Xu et al., (2019)	ANG2 mice	↔ PA	↘ Hypertrophy	ND
		↘ Fibrosis (TGFβ, COL1A1, COL3A1, FN, CTGF, SMA,FSP1)	↘ NPPA, NPPB	Systolic function
			↘ MYH7	not affected
			↔ FE	
*Ptpn1* ECKO Gogiraju et al. (2016)	TAC mice	↗ Capillary density	↗ FS	↗ Systolic function
		↗ Perfusion	↘ Hypertrophy	↗ Survival at 20 weeks
		↘ Hypoxia	↘ Lung weight	
		↗ KDR signaling		
		↘ Fibrosis (TGFβ, αSMA, COL3A1)		
		↘ Oxidative stress (NOX4)		
*Tp53* ECKO Gogiraju et al. (2015)	TAC female mice	↗ Capillary density	↗ FS	↗ Systolic function
		↘ Apoptosis (Caspase 3)	↘ Hypertrophy	↗ Survival at 24h
		↘ Hypoxia (CAIX)	↘ CM size	
		↔ EC dependent vasodilation		
		↘ Fibrosis (FSP1, COL1A1)		
*Lepr* ECKO Gogiraju et al. (2019)	TAC mice	↗ Capillary density	↗ FS	↗ Systolic function
		↘ EC apoptosis	↘ Hypertrophy	
		↗ mTOR-dependent autophagy	↘ NPPB	
		↘ Fibrosis	↗ MYH6/MYH7 ratio	
		↘ VCAM-1		
		↘ Inflammation (CD45)		
		↘ Oxidative stress (DHE)		
*Adk* ECKO Davila et al. (2020)	TAC mice	↗ EC dependent vaso dilation	↗ EF (+/-)	NS
			↘ CM size	
			↔ Hypertrophy	
*Capns1* ECKO Sun et al. (2020b)	Isoproterenol (5 mg/kg) S.C., 1 dose 1 week-post mice	↘ Fibrosis	↘ CM size	Improved cardiac remodeling

Nr3c2, Nuclear Receptor Subfamily 3 Group C Member 2; ECKO, endothelial cell specific knock out; ND, not determined; Scnn1a, Sodium Channel Epithelial 1 Subunit Alpha; Icam-1, Intracellular Cell Adhesion Molecule 1; FOXP1, Forkhead Box P1; Ets1, ETS Proto-Oncogene 1; Transcription Factor, Ptpn1**,** Protein Tyrosine Phosphatase Non-Receptor Type 1; Tp53, Tumor Protein P53; Lepr, Leptin receptor; Adk, Adenosine Kinase; Capns1, Calpain Small Subunit 1; DOC, deoxycorticosterone; HFD, High fat diet; MΦ, macrophages; TAC, transverse aortic constriction; ANG2, Angiotensin 2; IVRT, isovolumic relaxation time; MPI, myocardial performance index; CM, cardiomyocyte; EDP, end diastolic pressure; EF, ejection Fraction; FS, Fractional shortening; DHE, 2-hydroxy-ethidium.

Notably, inhibition of aldosterone signaling in ECs through endothelial specific disruption of Nuclear Receptor Subfamily 3 Group C Member 2 (*Nr3c2*) (Rickard et al., 2014; Jia et al., 2015) or endothelial specific disruption of Sodium Channel Epithelial 1 Subunit Alpha (*Scnn1a*) (Sowers et al., 2020) were shown to prevent diastolic dysfunction induced by a HFD, this was associated with decreased oxidative stress and/or inflammation (Jia et al., 2015; Sowers et al., 2020).

Also targeting endothelial activation by *Icam-1* KO was shown to prevent TAC-induced diastolic and systolic dysfunction (Salvador et al., 2016), this was associated with decreased inflammation and fibrosis.

Targeting TGFβ-induced signaling by overexpressing FOXP1 (Liu et al., 2019, 1) or disrupting ETS Proto-Oncogene 1, Transcription Factor (*Ets1*) specifically in ECs was shown to prevent ANG2-induced cardiac hypertrophy and diastolic dysfunction (Xu et al., 2019). As expected, this was associated with decreased cardiac fibrosis while arterial pressure was not modified.

Finally, promoting angiogenesis by endothelial specific disruption of Protein Tyrosine Phosphatase Non-Receptor Type 1 (*Ptpn1)* (Gogiraju et al., 2016), Tumor Protein P53 (*Tp53*) (Gogiraju et al., 2015) or *Lepr* (Gogiraju et al., 2019) was shown to improve TAC-induced cardiac hypertrophy and systolic dysfunction. This was associated with decreased cardiac hypoxia and fibrosis.

In conclusion, these series of experiments strongly support the fact that targeting EC dysfunction may be of interest to treat certain forms of HF and confirms the critical role of ECs in the pathophysiology of cardiac diseases. However, such strategies/targets remain to be tested in multi-hits models of HFpEF. Notably endothelial specific disruption of Adenosine Kinase (*Adk*) was shown to enhance endothelial dependent vasodilation and proposed to be protecting against TAC (Davila et al., 2020). Interestingly, the therapeutic potential of ADK inhibitors was tested in ZSF1 rats in which it was shown to improve diastolic dysfunction by decreasing EDP and Tau. This was not associated with significant reduction of cardiac hypertrophy (Davila et al., 2020).

#### Conclusion

Even though, clinical trials targeting NO signaling failed at demonstrating significant efficacy in patients with HFpEF, data obtained in preclinical studies are promising and further highlight the critical role of endothelial dysfunction in promoting cardiac oxidative stress, inflammation and fibrosis, three important features of HFpEF (Hage et al., 2020).

Whether or not empagliflozin improves cardiac function by targeting endothelial dysfunction is possible since it was shown to promote NOS3 phosphorylation, EC survival and quiescence. Besides, empagliflozin was shown to decrease oxidative stress, inflammation and cardiac fibrosis.

### EC Dysfunction May Not Cause Cardiomyocyte Impairment and Diastolic Dysfunction

Even though diastolic may be caused by endothelial dysfunction in the setting of HFpEF, the 2 other alternative hypotheses still have not been refuted. i.e., cardiomyocyte impairment observed in HFpEF may be independent on endothelial dysfunction: the 2 phenomenons occur concomitantly but are not related or cardiomyocyte impairment may cause endothelial dysfunction.

#### Cardiomyocyte Impairment Is Induced Independently on Endothelial Dysfunction

Recently, diastolic dysfunction was proposed to be initiated by cardiomyocyte impairment ahead of endothelial dysfunction in non-obese diabetic Goto-Kakizaki rats (Waddingham et al., 2019). Indeed, these rats were shown to develop diastolic dysfunction characterized by decreased dP/dt min and increased Tau, likely because of decreased cGMP levels and Titin N2B hypo-phosphorylation. Importantly, diastolic dysfunction in these rats was associated with oxidative stress and elevated IL-6, TGFβ1 and NOX2 levels while endothelium dependent vasodilation was not altered. However, in this study the authors have not tested any of the other endothelial properties, notably EC activation, survival, paracrine activity.

Another study shows that in mice in which HFpEF is induced by a HFD regimen conjugated with continuous L-NAME administration, diastolic dysfunction is consecutive to increased NOS2 expression in cardiomyocytes (Schiattarella et al., 2019). More specifically, increased NO production following NOS2 overexpression would promote S-nitrosylation of IRE1α leading to decreased *Xbp1* splicing. Decreased levels of spliced XBP1 (sXBP1) decreases ubiquitination and proteasomal degradation of FOXO1 by E3 ubiquitin ligase STUB1 (STIP1 homology and U-box-containing protein 1) inducing FOXO1 accumulation in cardiomyocytes (Schiattarella et al., 2019; 2021). However, it is not known whether inhibition of NOS2 prevents capillary rarefaction and decreased coronary flow reserve observed in this model.

#### Cardiomyocyte Impairment May Cause Endothelial Dysfunction

Signals produced by Cardiomyocyte have been shown by control maintenance and growth of cardiac vasculature. Notably, specific disruption of *Vegfa* in cardiomyocyte was shown to decrease capillary density, in the heart (Giordano et al., 2001). Similarly, cardiomyocyte signal transducer and activator of transcription 3 (STAT3) was proposed to promote vascular formation in the heart since cardiac-specific activation of STAT3 was shown to promotes vascular formation in the heart (Osugi et al., 2002) while cardiomyocyte-specific disruption of *Stat3* was shown to be associated with decreased cardiac capillary density (Hilfiker-Kleiner et al., 2004).

More recently, cardiomyocyte to ECs signaling was also shown to regulate endothelium integrity and function since mice with cardiomyocyte specific overexpression of G Protein Subunit Alpha Q (*Gnaq*) display increased capillary leakage (from 8 months of age) and impaired endothelial-dependent vasodilation (from 10 months of age). This is proposed to be due to an alteration of the red blood cell phenotype, notably mice with cardiomyocyte specific overexpression of GNAQ had smaller and stiffer red blood cells (Mohaissen et al., 2021).

## Discussion

First, this review highlights that even though HFpEF is known to be associated with vascular and microvascular dysfunction for 10 years, the proposed causal role of endothelial dysfunction in the pathophysiology of this disease is challenging to demonstrate. First, animal models of HFpEF have been difficult to define. Second, the role/phenotype of the cardiac vasculature is often neglected in studies investigating the pathophysiology of HF. Third, demonstrating the specific role of endothelial dysfunction in the pathophysiology of HFpEF requires the use of tissue-specific conditional KO mice.

Importantly, this revue demonstrates the critical role of ECs in cardiac physiology through the results of studies in which the authors have assessed the cardiac phenotype of mice or rats with specific disruption/overexpression of genes in ECs. Notably, these studies show that modifications of endothelial properties may induce both systolic and diastolic dysfunction. The modifications directly affecting the metabolism or growth of cardiomyocyte seems to induce systolic dysfunction while the modification of EC properties leading to increased oxidative stress was shown to induce diastolic dysfunction without inducing cardiomyocyte hypertrophy. Notably, cardiac hypertrophy is not an obligatory feature of HFpEF since 31% of patients with HFpEF do not have cardiac hypertrophy (Shah and Pfeffer, 2012).

At molecular and cellular level, this revue shows that the endothelial to cardiomyocyte crosstalk is still poorly understood, and that some paradigm needs to be carefully taken into account. Notably, the proposed role of NO, NRG1, APLN and EDN1 in mediating endothelial to cardiomyocyte signaling is more likely not true since these molecules mainly acts autocrinally (Shohet et al., 2004; Dugaucquier et al., 2020; Segers and De Keulenaer 2021). Besides, the role of endothelial to mesenchymal transition often seen *in vitro* and proposed to contribute to cardiac fibrosis needs to be carefully taken into account since this phenomenon more likely do not exist in the adult heart (Moore-Morris et al., 2014).

In conclusion, the role of ECs in the pathophysiology of HF is still far from being understood, the use of the recently described multi-hits models of HFpEF in combination with endothelial specific KO mice and a better characterization of the microvascular phenotype should bring some clues regarding HFpEF pathophysiology and identify new therapeutic targets.

## Glossary

AAC: ascending aortic constriction
AAV: adeno-associated virus
ADAMTS13: a disintegrin and metalloprotease with thrombospondin type I repeats-13
ADRB3: β3-adrenergic receptor
AKT1: AKT Serine/Threonine Kinase 1
AMPK: AMP-activated protein kinase
ANG2: angiotensin 2
ANGPTL4: angiopoietin-like 4
APLNR: apelin Receptor
ATP2A2: ATPase Sarcoplasmic/Endoplasmic Reticulum Ca2+ Transporting 2
CAMK2: calcium/calmodulin-dependent protein kinase II
Cch: carbamoylcholine
CCL2: chemokine (CC motif) ligand 2
CDH5: cadherin 5
CFR: coronary flow reserve
cGMP: cyclic guanosine monophosphate
CMR: cardiovascular magnetic resonance
CTGF: connective tissue growth factor
CYBA: cytochrome B-245 Alpha Chain
CXCL8: C-X-C Motif Chemokine Ligand 8
DOCA: deoxycorticosterone acetate
DOCP: desoxycorticosterone pivalate
EC: endothelial cell
EDP: end-diastolic pressure
EF: ejection fraction
EndoMT: endothelial cells into mesenchymal cells transition: endothelial to mesemchymal transition
eNOS: endothelial Nitric Oxide Synthase
EphB4: EPH Receptor B4
EphB4ECKO: EPH Receptor B4 endothelial cell knockout
EDN1: endothelin
ERBB2: Erb-B2 Receptor Tyrosine Kinase 2
ERBB4: Erb-B2 Receptor Tyrosine Kinase 4
EndoMT: endothelial cells into mesenchymal cells transition: endothelial to mesemchymal transition
FABP4/5: fatty Acid Binding Protein 4/5
FA: fatty acid
Foxp1: forkhead box protein P1
FoxO1: forkhead box protein O1
FS: fractional shortening
FLT1: fms Related Receptor Tyrosine Kinase 1
GPCR: G protein-coupled receptor
GRK: G protein-coupled receptor kinase
H_2_O_2_: hydrogen peroxide
Hif1a: hypoxia-inducible factor 1-alpha
HF: heart failure
HFD: high fat diet
HFpEF: heart failure with preserved ejection fraction
HFmrEF: heart failure with mid-range ejection fraction
HFrEF: heart failure with reduced ejection fraction
HLA: histocompatibility leukocyte antigen
ICAM-1: intercellular adhesion molecule-1
IL-1: interleukin-1
IL-1β: interleukin-1β
IL-6: interleukin-6
IL-10: interleukin-10
IL-18: interleukin-18
IVRT: isovolumic Relaxation Time
IVSD: interventricular septum at end diastole
KDR: kinase Insert Domain Receptor
LAD: left anterior descending artery
Lepr^db/db^: leptin receptor deficient db/db mice
LIPG: endothelial lipase
L-NAME: N(ω)-nitro-L-arginine methyl ester
LV: left ventricle
LVEDP: left ventricular end-diastolic pressure
LVEF: left ventricular ejection fraction
LYVE1: lymphatic vessel endothelial hyaluronan receptor 1
MYH7: myosin heavy chain 7
MAPK14: mitogen-Activated Protein Kinase 14 = P38
MAPK8: mitogen-Activated Protein Kinase 8 = JNK
NET: neutrophil extracellular traps
NF-κB: nuclear factor-kappa B
NO: nitric Oxide
NOS1: nitric Oxide Synthase 1
NOS2: nitric Oxide Synthase 2
NOS3: nitric Oxide Synthase 3
NOX2: nicotinamide adenine dinucleotide phosphate oxidase-2
NOX4: nicotinamide adenine dinucleotide phosphate oxidase-4
MPI: myocardial Performance Index
NRG1: neuregulin 1
NTproBNP: N-terminal prohormone of brain natriuretic peptide
NPPA: natriuretic Peptide A = ANP
NPPB: natriuretic Peptide B = BNP
Nr3c2: nuclear Receptor Subfamily 3 Group C Member 2 = Mineralocorticoid receptor
O_2_^−^: superoxide anion
O_2_: dioxygen
PDZRN3: PDZ Domain Containing Ring Finger 3,
PECAM1: platelet and endothelial cell adhesion molecule 1
PRKG1: protein Kinase cGMP-Dependent 1
PRKA: protein Kinase cAMP-Activated
PGF: placental growth factor
PRSW: preload recruitable stroke work
PROX1: prospero homeobox protein 1
PLN: phospholamban
Rbpj: recombination signal binding protein for immunoglobulin kappa J region
RbpjECKO: recombination signal binding protein for immunoglobulin kappa J region endothelial cell knockout
ROS: reactive oxygen species
S1pr1: sphingosine-1-phosphate receptor 1
SELE: E-selectin
Sirt1: sirtuin 1
Sirt1 ECKO: sirtuin 1 endothelial cell knockout
Sirt3: sirtuin 3
Sirt3^ECKO^: sirtuin 3 endothelial cell knockout
sGC: soluble guanylate cyclase
SHHR: spontaneously hypertensive heart failure rat
SNP: sodium nitroprusside
SR: sarcoplasmic reticulum
STAT3: signal transducer and activator of transcription 3
STUB1: STIP1 homology and U-box-containing protein 1
sXbp1: spliced X-box binding protein 1
SLC2A1: solute Carrier Family 2 Member 1 = GLUT1
SLC2A4: solute Carrier Family 2 Member 4 = GLUT4
SAMP8: senescence accelerated mouse prone 8
Scnn1a: sodium Channel Epithelial 1 Subunit Alpha
TAC: transverse aortic constriction
TCRα: T-cell receptor α
TGFβ1: transforming growth factor beta 1
TNF: tumor necrosis factor
VCAM-1: vascular cell adhesion protein 1
VEGFA: vascular endothelial growth factor A
VwF: von Willebrand factor
VEGFC: vascular Endothelial Growth Factor C
WD: western diet
ZDF: zucker Diabetic Fatty
ZSF1: zucker fatty/Spontaneously hypertensive heart failure F1 hybrid
